# Apelin is expressed in intimal smooth muscle cells and promotes their phenotypic transition

**DOI:** 10.1038/s41598-023-45470-z

**Published:** 2023-10-31

**Authors:** Luís Miguel Cardoso Dos Santos, Pascal Azar, Cécile Brun, Stéphane König, Angela Roatti, Alex J. Baertschi, Chiraz Chaabane, Marie-Luce Bochaton-Piallat

**Affiliations:** 1 Department of Pathology and Immunology, University of Geneva, Geneva, Switzerland; 2 Geneva University Hospitals, University of Geneva, Geneva, Switzerland; 3 Department of Neuroscience, University of Geneva, Geneva, Switzerland; 4 Department of Physiology and Metabolism, Faculty of Medicine, University of Geneva, Geneva, Switzerland

**Keywords:** Atherosclerosis, Restenosis

## Abstract

During atherosclerotic plaque formation, smooth muscle cells (SMCs) switch from a contractile/differentiated to a synthetic/dedifferentiated phenotype. We previously isolated differentiated spindle-shaped (S) and dedifferentiated rhomboid (R) SMCs from porcine coronary artery. R-SMCs express S100A4, a calcium-binding protein. We investigated the role of apelin in this phenotypic conversion, as well as its relationship with S100A4. We found that apelin was highly expressed in R-SMCs compared with S-SMCs. We observed a nuclear expression of apelin in SMCs within experimentally-induced intimal thickening of the porcine coronary artery and rat aorta. Plasmids targeting apelin to the nucleus (N. Ap) and to the secretory vesicles (S. Ap) were transfected into S-SMCs where apelin was barely detectable. Both plasmids induced the SMC transition towards a R-phenotype. Overexpression of N. Ap, and to a lesser degree S. Ap, led to a nuclear localization of S100A4. Stimulation of S-SMCs with platelet-derived growth factor-BB, known to induce the transition toward the R-phenotype, yielded the direct interaction and nuclear expression of both apelin and S100A4. In conclusion, apelin induces a SMC phenotypic transition towards the synthetic phenotype. These results suggest that apelin acts via nuclear re-localization of S100A4, raising the possibility of a new pro-atherogenic relationship between apelin and S100A4.

## Introduction

Apelin was first discovered in 1998 by Tatemoto and colleagues, and identified as an endogenous ligand for the orphan G protein-coupled receptor APJ^1^. It is distributed both in the central nervous system and peripheral tissues of humans and rodents. Apelin is widely and abundantly expressed in the cardiovascular system^2^. In particular, it is expressed by endothelial and vascular smooth muscle cells (SMCs)^3^. Apelin regulates vascular tone both as an endothelium-dependent vasodilator, through nitric oxide production, and as an endothelium-independent vasoconstrictor, by acting directly on the APJ receptor of SMCs^4,5^. Besides, apelin peptide plays a crucial role in neovascularization of ischemic tissue and angiogenesis^6^.

The apelin gene encodes a 77 amino acid preproapelin that contains a N-terminal signal peptide. Proapelin is processed into C-terminal fragments giving rise to several apelin isoforms, such as apelin-13, -17, -36 as well as the pyroglutamate form of apelin-13, [Pyr^1^] apelin-13^7^. In human heart, apelin-13 has been found to be the predominant isoform^8^ whereas in the plasma, apelin-17 and, to a lesser extent apelin-13, are predominant^9^. Various proteases are thought to regulate apelin cleavage and isoform generation but, to date, only the proprotein convertase subtilisin/kexin 3 (PCSK3) has been clearly described to directly cleave apelin-55 into apelin-13^10^. Although angiotensin-converting enzyme type 2 has been proposed as one of the enzymes involved in the processing of apelin isoforms^11^, it is more likely to be implicated in the inactivation of the peptide by removing the C-terminal phenylalanine and reducing its biological effects^12^. Thus, the mechanisms regulating the secretory pathway and post-translational processing of apelin peptides are still not clear.

Studies performed with ApoE-deficient mice have led to contradictory results. Knocking out apelin in ApoE-deficient mice resulted in accelerated atherosclerosis development^13^, suggesting an anti-atherogenic role for apelin. In contrast, depletion of the APJ receptor in ApoE-deficient mice decreased atherosclerotic lesion progression^14^, potentially indicating that apelin is a pro-atherogenic molecule. Likewise, in carotid ligation-induced intimal thickening, neointimal lesion is decreased in apelin-deficient mice as compared to the wild-type mice, an effect which is abrogated by infusion of apelin^15^. In humans, apelin was shown to be upregulated in atherosclerotic plaque, co-localizing with markers for macrophages and SMCs^4^. In plasma, apelin is negatively correlated both with the progression of the pathology in atherosclerotic patients, and with the increase of LDL levels, compared with healthy controls^16,17^. Mechanistically, several pathways have been observed to be affected by the apelin/APJ system, which appears to be isoform dependent. Of particular interest, it has been shown that apelin-13 is capable of inducing SMC proliferation, through Cyclin D1^18^, and migration, partly by promoting matrix metalloproteinase (MMP)-2 activation^19^, both considered critical processes in the pathogenesis of atherosclerosis. Nonetheless, it is still unclear whether apelin exerts anti- or pro-atherogenic effects, and which pathways could be involved in these observed effects.

We established a model of SMCs, in which we were able to isolate, from normal porcine coronary arteries, two phenotypically distinct populations of SMC: spindle-shaped (S) and rhomboid (R) SMCs^20^. While the first one is typical of the contractile SMC phenotype and represents the predominant population in the normal media of arteries, the latter is typical of the synthetic SMC phenotype, and exhibits dedifferentiated features associated with increased proliferation and migration. We have also demonstrated that by treating S-SMCs with platelet-derived growth factor (PDGF)-BB, a well-known factor responsible for SMC dedifferentiation, we were able to achieve a complete transition toward the synthetic phenotype (R-SMCs). Moreover, we identified S100A4, a Ca^2+^-binding protein, as a marker of the R-SMCs in vitro in the porcine coronary artery and in intimal SMCs in vivo in mice^21^, pigs, and humans^22^. Thus, it has been hypothesized that S100A4 could play a role in atherosclerotic plaque development. Recently, we have shown that the extracellular form of S100A4 is essential for the SMC phenotypic transition^23^ and acts through toll-like receptor-4 (TLR-4)^21^.

In this study, we tested the possible role of intracellular apelin fragments in porcine SMC function and phenotypic transition, a process typical of atherosclerotic plaque formation, and its relationship with S100A4.

## Results

### Endogenous apelin is expressed in intimal SMCs

**Figure 1:**
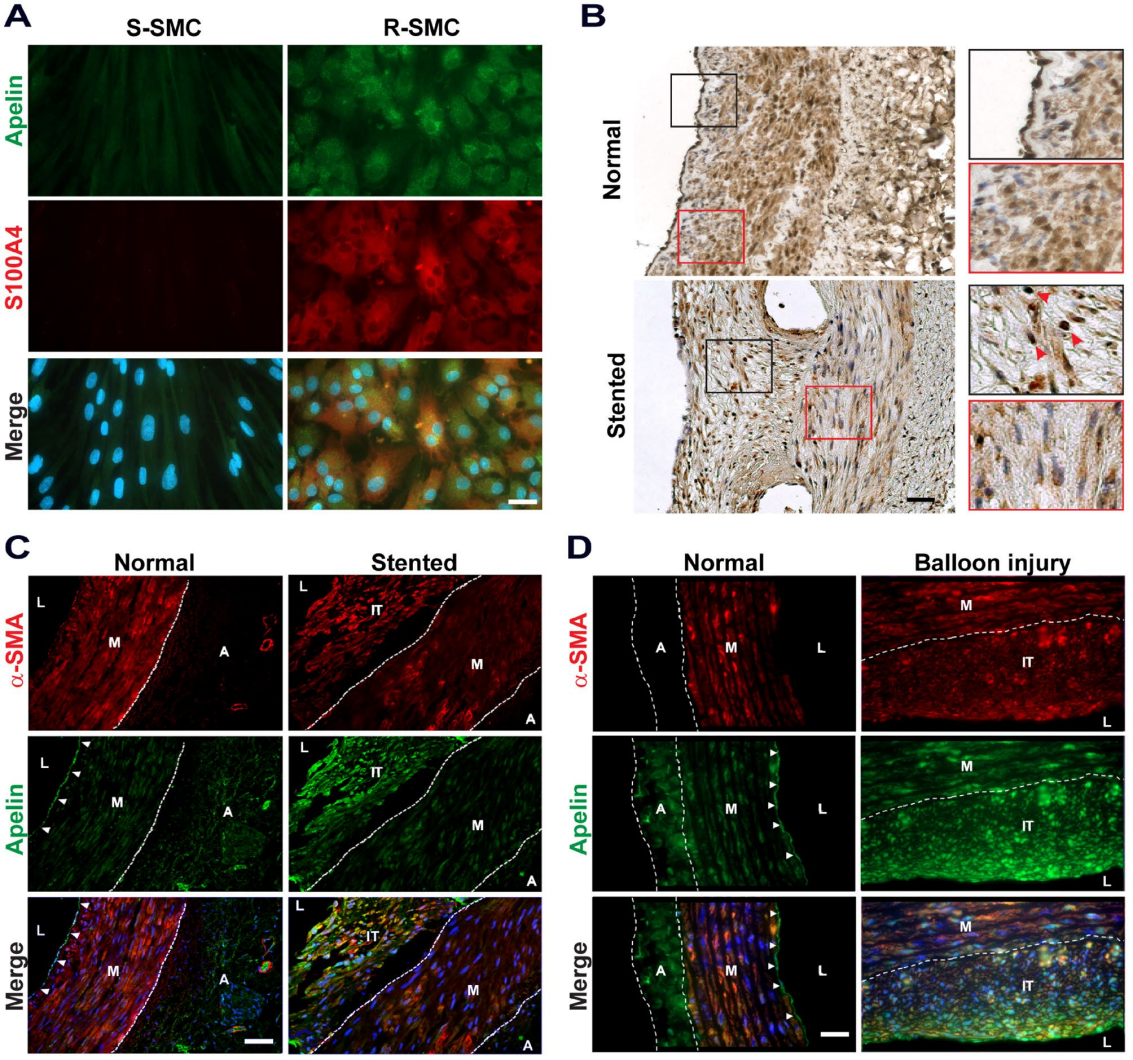
Expression of apelin in vitro in S- and R-SMCs and in vivo in porcine coronary artery after stent-induced intimal thickening and rat aorta after balloon-induced intimal thickening. (**A**) Double immunofluorescence staining showing apelin and S100A4 expression in S-SMCs and R-SMCs, nuclei are stained in blue with DAPI, Scale bar = 30μm. (**B**) Immunohistochemistry showing expression of apelin in normal and stented porcine coronary artery. Black and red squares indicate 8 × increased magnification. Red arrowheads indicate apelin expression in the nucleus, Scale bar = 20μm (n = 2 for each group). (**C, D**) Double immunofluorescence staining showing expression of α-SMA (red) and apelin (green) in normal and stented porcine (**C**) coronary artery (n = 2 for each group) as well as rat aortas from control (n = 3) and balloon-induced intimal thickening (n = 5, **D**). Nuclei are stained in blue with DAPI. Dotted lines highlight the media and adventitia. Arrowheads indicate the endothelium. Scale bar = 100μm. L, lumen; IT, intimal thickening; M, media; A, adventitia.

In vitro*,* we showed that apelin was strongly expressed in R-SMCs whereas it was practically absent in S-SMCs (Fig. 1A). S100A4 was present in R-SMCs and hardly detectable in S-SMCs, as previously described^22^. In vivo*,* we analyzed the expression of apelin in porcine and rat SMCs. Immunohistochemistry performed on healthy porcine coronary artery specimens showed that apelin was strongly expressed in endothelial cells (ECs, Fig. 1B, top panel, black square), whereas its expression was weaker in the SMCs of the media (top panel, red square). After stent implantation, apelin was strongly expressed in the nuclei of intimal SMCs (Fig. 1B, bottom panel, black square) whereas its expression in the SMCs of the underlying media was weaker and mainly cytoplasmic (Fig. 1B, bottom panel, red square). The cytoplasmic apelin staining is weaker in the media of stented arteries compared to normal arteries. These results were confirmed by double immunofluorescence staining for apelin and α-smooth muscle actin (α-SMA), a marker of SMC differentiation. Apelin was strongly expressed in ECs of normal porcine (Fig. 1C) and rat (Fig. 1D) arteries as well as in non-lesional areas of balloon-induced intimal thickening of rat arteries. Moreover, apelin was co-expressed with α-SMA in intimal cells after stent implantation or balloon injury (Fig. 1C,D, respectively) whereas it was hardly detectable in medial SMCs (Fig. 1C). Co-staining with DAPI confirmed that apelin was expressed in nuclei of intimal SMCs. Therefore, apelin is overexpressed in intimal SMCs in pigs and rats and exhibits an unexpected tropism for nuclei.

### Characterization of mutated preproapelin (eGFP/HisTag) fusion protein

**Figure 2:**
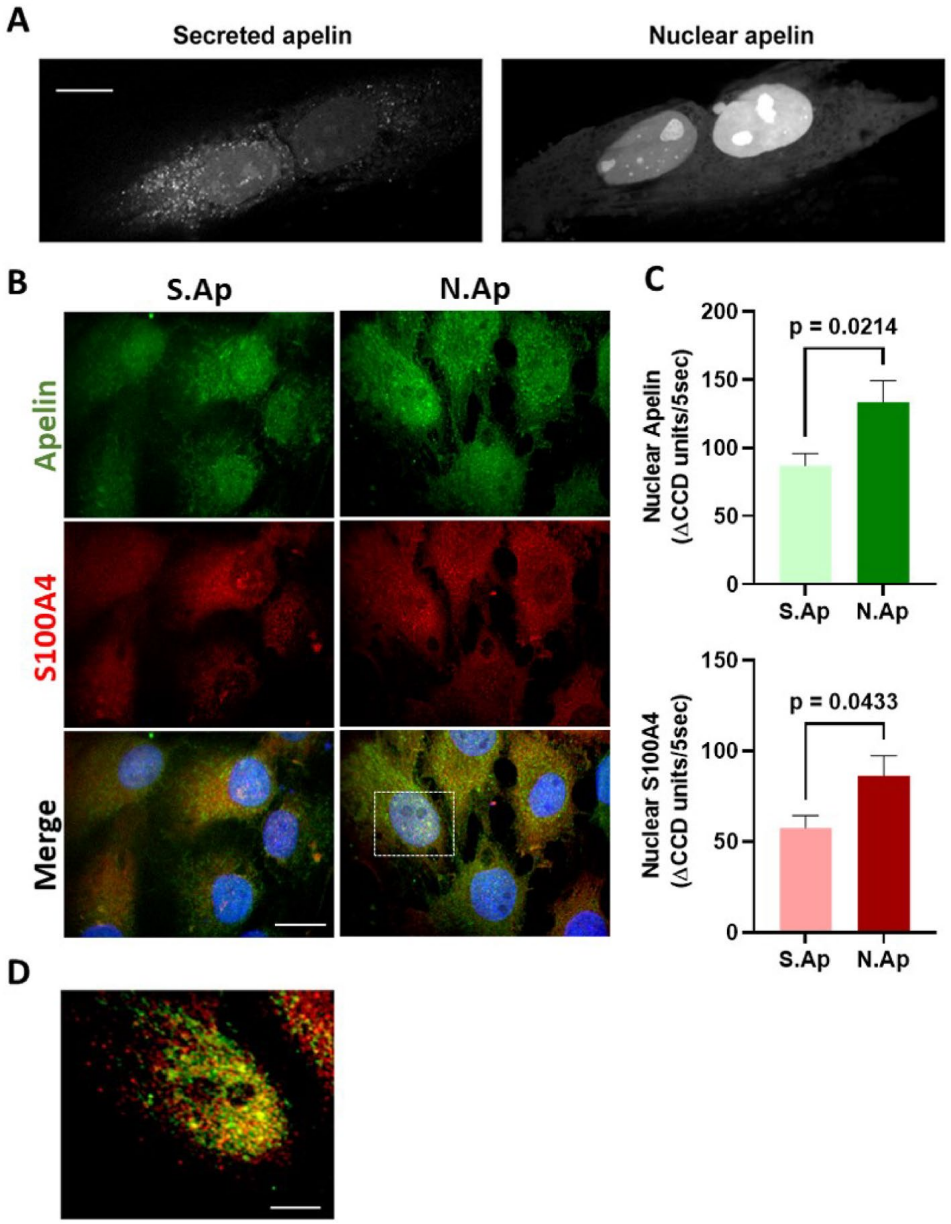
Overexpression of apelin plasmids and colocalization with S100A4 in SMCs. (**A**) Expression of mutated apelin/eGFP constructs is observed by live imaging of SMCs: Scale bar = 10 μm. Representative confocal microscopy staining (**B**) and nuclear quantification (**C**) of apelin and S100A4 expression in S-SMCs 96 h after transfection with S.Ap (n = 23 nuclei) or N. Ap (n = 29 nuclei)-encoded plasmids. Nuclei are stained in blue with DAPI. Scale bar = 10 μm. (**D**) 2.5 × magnified region highlighted by the dashed square in (**B**) and showing apelin and S100A4 expression in the nucleus. Scale bar = 4 μm.

Because apelin showed a tropism toward SMC nuclei, we generated two different variants of apelin. By mutating the preproapelin cDNA at the first or second ATG site, we modified the propensity of apelin to be either targeted to the nucleus or to the secretory vesicles, respectively (see Methods). Both variants of the mutated preproapelin were inserted in a pEGFP-N1 plasmid and subsequently transfected into S-SMCs. Expression of mutated preproapelin/eGFP fusion protein was observed by live imaging of transfected S-SMCs: S. Ap-GFP was found in fast moving vesicles whereas the N. Ap-GFP appeared in nucleolus and dense dots that resemble nuclear bodies with both variants inducing a transition of S-SMCs towards R-SMCs (Fig. 2A). The S- to R- transition was achieved even when transfecting S-SMCs with the empty pEGFP-N1 plasmid (data not shown). To address this issue, we changed the backbone of the two mutated preproapelin forms by exchanging the eGFP marker with a His-tag, followed by the insertion of each one of the mutated preproapelin into a pcDNA3.1 vector (see methods). To confirm the nuclear localization of apelin expression after transfection with N.Ap-encoded His-tag expressing plasmid in S-SMCs, we performed confocal microscopy. We confirm that N.Ap leads to a higher nuclear expression of apelin compared to S.Ap (Fig. 2B and C). We found, unexpectedly, that the nuclear expression of apelin was associated with an increased nuclear expression of S100A4 (Fig. 2B,C and D).

### N. Ap and S. Ap overexpression in S-SMC induces SMC phenotypic transition, proliferation and S100A4 release

**Figure 3:**
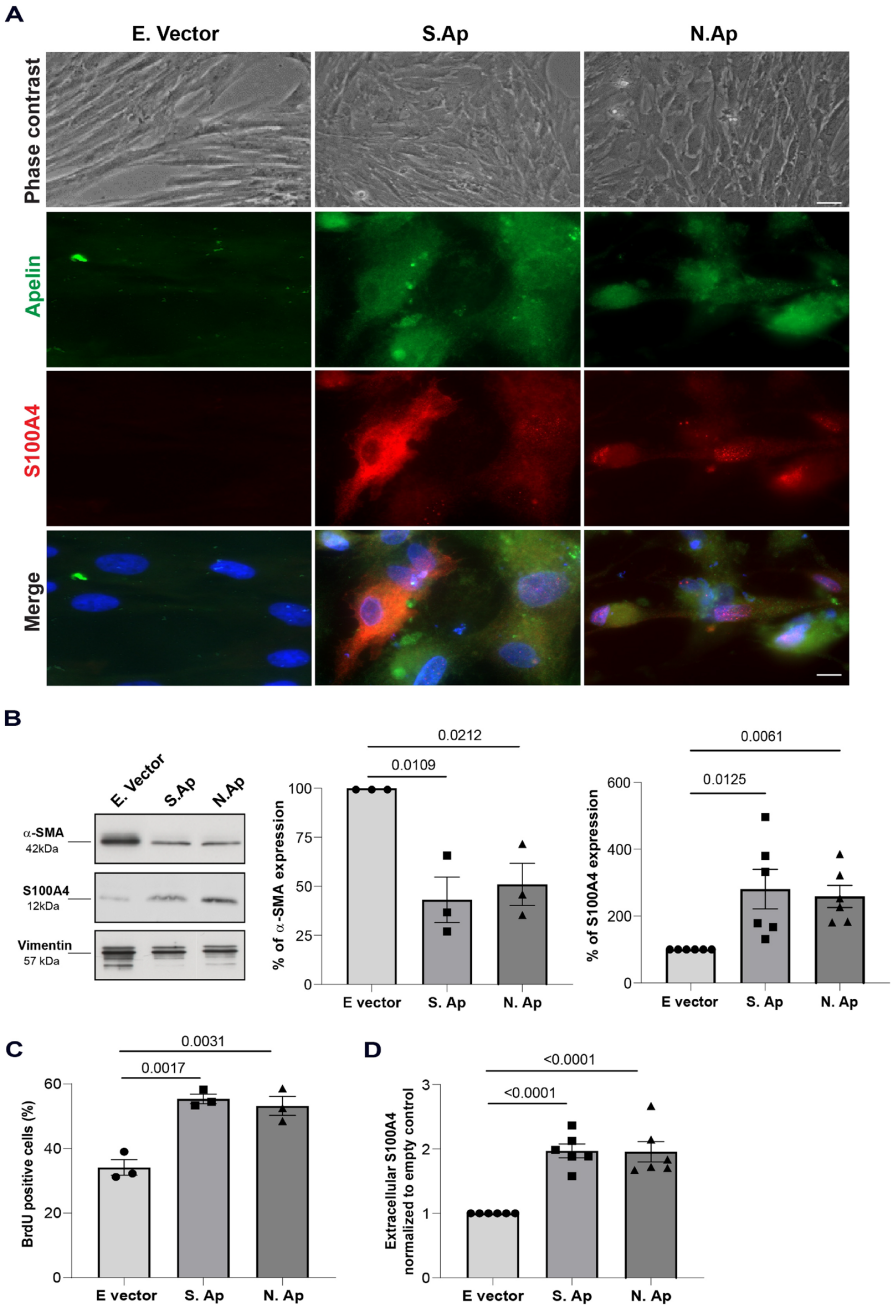
Effect of S. Ap and N. Ap overexpression in SMC phenotypic transition. (**A**) Representative phase-contrast photomicrographs (scale bar = 20 μm) and double immunofluorescence staining (scale bar = 10 μm) showing apelin and S100A4 expression in empty vector-transfected, S. Ap-transfected and N. Ap-transfected S-SMCs. (**B**) Representative immunoblot (cropped images showing important bands only) and bar graph showing α-SMA and S100A4 expression (n = 3 and n = 6, respectively) normalized to empty vector in transfected S-SMCs 96 h after transfection with empty vector (dots), S. Ap (squares), and N. Ap (triangles), original blots are presented in pages 7–9 of the supplementary file. (**C**) Bar graph showing the percentage of BrdU-positive S-SMCs, 96 h after transfection with empty vector (dots), S. Ap (squares), and N. Ap (triangles, n = 3). (**D**) Bar graph showing extracellular S100A4 levels detected by competitive ELISA assay in S-SMC supernatants after transfection with empty vector (dots), S. Ap (squares), and N. Ap (triangles, n = 6).

We confirmed that transfection of the empty pcDNA3.1 vector did not affect the SMC phenotype (Fig. 3A top panel). We then proceeded by overexpressing the mutated preproapelin/His-tag fusion proteins (S. Ap and N. Ap) in S-SMCs, in which apelin was barely or not detectable. Interestingly, S-SMCs transfected with S. Ap or N. Ap acquired a R-phenotype 96 h after transfection and expressed S100A4 (Fig. 3A). Moreover, S. Ap transfection in S-SMCs induced a more prominent S100A4 cytoplasmic expression, whereas N. Ap transfection led to a higher S100A4 nuclear expression (Fig. 3A). As expected, S-SMCs transfected with the empty vector maintained their S-phenotype and did not express S100A4 nor apelin (Fig. 3A). Western blot analysis of protein extracts for the same conditions confirmed that S. Ap and N. Ap overexpression induced significant decrease of α-SMA (43.12 ± 11.62% and 50.96 ± 10.77%, respectively; *P* = 0.0109 for S. Ap and *P* = 0.0212 for N. Ap, n = 3). This decrease was associated with the synthetic/dedifferentiated phenotype, and with an increase in S100A4 expression (280.8 ± 59.5% and 258.5 ± 80.9%, respectively; *P* = 0.0125 for S. Ap and *P* = 0.0061 for N. Ap, n = 6), compared with empty vector transfection (Fig. 3B). In addition, at 96 h, S. Ap and N. Ap-transfected cells exhibited enhanced BrdU incorporation compared with empty vector-transfected cells (55.4 ± 1.5% and 53.2 ± 3.0% vs 34.2 ± 2.5%, respectively; *P* = 0.0017 for S. Ap and *P* = 0.0031 for N. Ap; n = 3, Fig. 3C), indicating that S. Ap and N. Ap overexpression is associated with increased SMC proliferation.

We have previously demonstrated that extracellular S100A4 is responsible for SMC phenotypic transition. For this purpose, we performed competitive ELISA assay^23^, and confirmed that the levels of extracellular S100A4 were higher in the supernatants of S. Ap and N. Ap-transfected S-SMCs than in empty vector-transfected S-SMCs (ELISA values were normalized and expressed as fold increase in comparison with empty vector control values set at 1, *P* < 0.0001 for both; n = 6; Fig. 3D). These results suggest that N. Ap and S. Ap transfection in differentiated/contractile S-SMCs induces a transition towards a R-phenotype associated with increased proliferative activity, downregulation of α-SMA expression, and increased expression and release of S100A4, typical markers of the dedifferentiated/synthetic phenotype.

### Expression of apelin is correlated to SMC phenotypic transition

**Figure 4:**
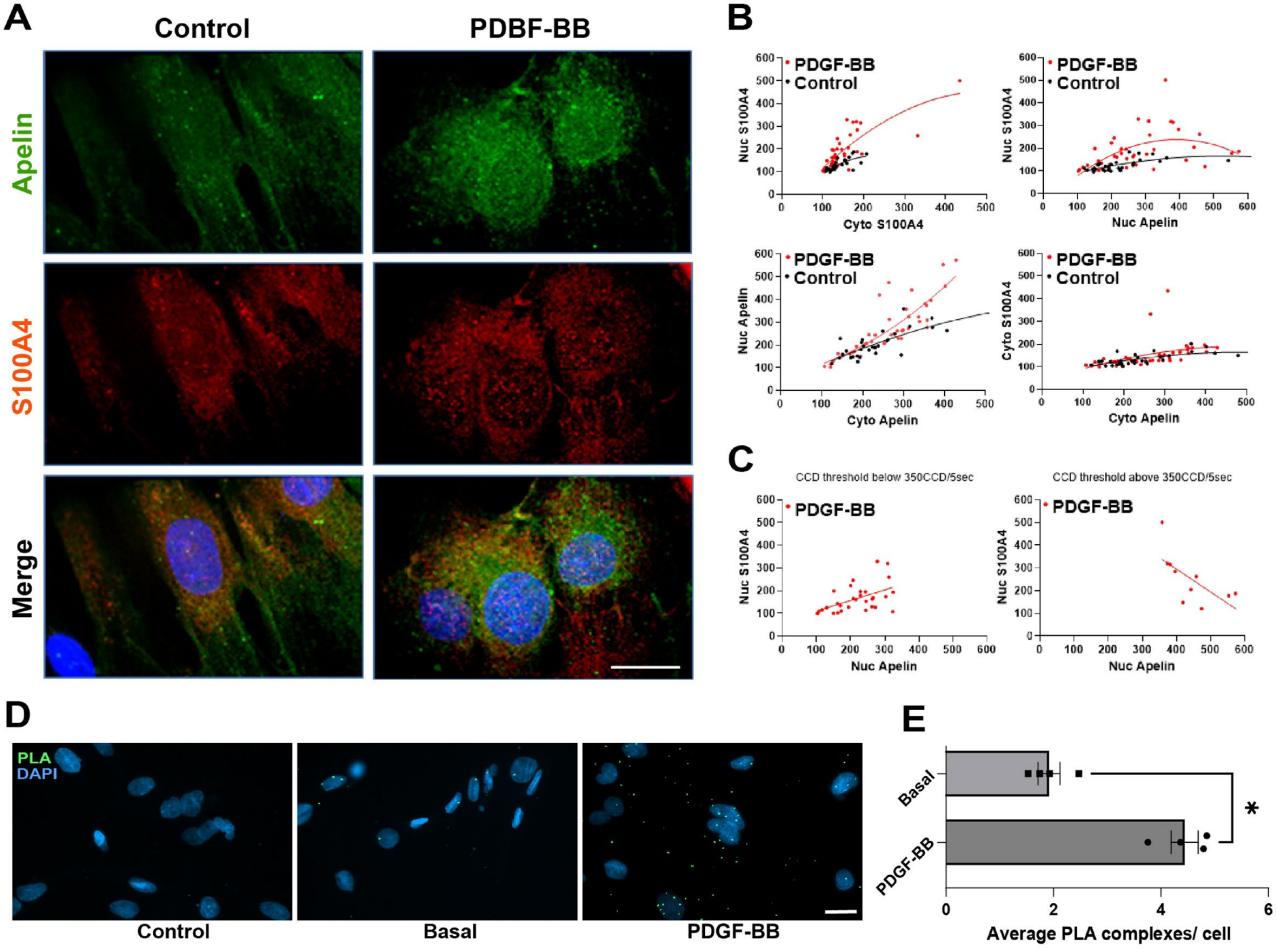
PDGF-BB mediated SMC phenotypic switch induces interaction and nuclear expression of apelin and S100A4. (**A**) Double immunofluorescence staining showing apelin, S100A4, and both in S-SMCs after treatment with PDGF-BB for 96 h (n = 4). Nuclei are stained in blue with DAPI. Scale bar = 10 μm. (**B**) Graphs showing quantification of nuclear versus cytoplasmic apelin and S100A4 in S-SMCs treated with PDGF-BB vs control. Black and red lines were generated from a second order polynomial non-linear fit regression. (**C**) Data were separately displayed for a threshold value below or above 350 CCD units/5 s, and were analysed by simple linear regression and Pearson’s correlation. (**D**) Representative images of apelin and S100A4 interaction detected by in situ proximity ligation assay (PLA) in non-stimulated (Basal) and PDGF-BB stimulated SMCs (PDGF-BB). The control condition represents the absence of one of the primary antibodies (here the rabbit anti-apelin, Control). Scale bar, 20 μm. (**E**) Statistical analysis of average PLA complexes per cell (n = 4), Mann–Whitney non-parametric test, *p = 0.0286.

Since apelin staining was only observed in intimal SMCs, we investigated whether apelin was restricted to SMCs that underwent a transition from the contractile (S-) to the synthetic (R-) phenotype. For this purpose S-SMCs were treated with PDGF-BB, which is known to induce the SMC phenotypic changes, resulting in, among others, increased proliferation and migratory properties, decreased expression of differentiation markers and increased expression of S100A4^20,22,23^. Treatment of S-SMCs with PDGF-BB yielded a marked upregulation of apelin and S100A4 compared with control S-SMCs (Fig. 4A). Of note, the increased S100A4 expression in S-SMCs upon PDGF-BB stimulation is lower than in R-SMCs^23^. Moreover, we observed a nuclear expression of apelin in cells treated with PDGF-BB, which corroborated the in vivo results. Interestingly, we also demonstrated that apelin and S100A4 expression increased proportionally. Quantification of nuclear versus cytoplasmic apelin (coefficient of determination R^2^ = 0.617, n = 30 cells, *p* < 0.0001, slope = 0.572 ± 0.083 in control and R^2^ = 0.709, n = 40 cells, *p* < 0.0001, slope = 1.16 ± 0.12 in PDGF-BB) and nuclear versus cytoplasmic S100A4 (R^2^ = 0.58, n = 30 cells, *p* < 0.0001, slope = 0.732 ± 0.111 in control and R^2^ = 0.602, n = 40 cells, *p* < 0.0001, slope = 1.06 ± 0.14 in PDGF-BB) showed that PDGF-BB increased the nuclear expression of both apelin and S100A4 (Fig. 4B, left panels). The quantification of nuclear apelin versus nuclear S100A4 (R^2^ = 0.358, n = 30 cells, p = 0.0002, slope = 0.123 ± 0.029 in control and R^2^ = 0.157, n = 40 cells, *p* = 0.0113, slope = 0.28 ± 0.1 in PDGF-BB) confirmed the nuclear localization and co-expression of both apelin and S100A4 in PDGF-BB-treated S-SMCs compared with control cells (Fig. 4B, right panels). Setting a threshold of apelin quantification at 350 CCD (corresponding to the apex of the red hyperbolic line in Fig. 4B upper right panel) further revealed a duality in the interaction between apelin and S100A4. At higher expression levels of apelin, S100A4 did not localize in the nuclei anymore (Fig. 4C, R^2^ = 0.254, n = 30 cells, *p* = 0.0044, slope = 0.466 ± 0.15 below 350 CCD units/5 s and R^2^ = 0.443, n = 10 cells, *p* = 0.035, slope = −1.006 ± 0.39 above 350 CCD units/5 s). To further decipher the relationship between apelin and S100A4, we performed proximity ligation assay (PLA) to detect possible protein–protein interactions. Upon PDGF-BB stimulation, PLA apelin/S100A4 complexes were detected in both the nuclei and cytoplasm of SMCs (Fig. 4D and E). These results suggest that apelin plays a role in the phenotypic transition of SMCs by affecting S100A4 expression/localization.

## Discussion

It is well defined that the phenotypic transition of SMCs from a contractile to a synthetic phenotype is a physiological response to repair the vessel from an injury^24^. The model of stent implantation we used mimics a typical case of restenosis, where the intimal thickening is mainly composed of SMCs. We found apelin to be expressed in SMCs that underwent a phenotypic transition and migrated towards the intima, from the media layer, which stained negative for apelin. Upon double immunofluorescent staining, we observed that the majority of apelin positive cells are also positive for α-SMA. This agrees with other studies that showed significantly increased apelin levels in human atherosclerotic plaques^4,19^, and that these cells were positive for α-SMA^19^. Such specific expression delineates a crucial role for apelin in the phenotype of SMCs responsible for plaque formation and progression. It is noteworthy that SMCs lose the expression of their usual contractile markers after switching their phenotype^24–27^. Thus their contribution to the heterogeneity of the cellular content of atherosclerotic plaque has been underestimated, as recently described in lineage tracing mouse models^28,29^. In this context, a broad marker of intimal SMCs is still missing and our results reveal apelin as a possible novel marker of those SMCs.

Apelin has been widely described as the endogenous ligand of the APJ surface receptor through which it regulates various physiological processes^1^, but to the best of our knowledge, there is no report of apelin being addressed to the nucleus. We found that apelin has a tropism towards the nuclei of intimal SMCs that led us to investigate this expression by modulating the intracellular trafficking of the peptide. Our results show that an alternative translation initiation site is able to address apelin towards the nucleus where it is co-expressed with S100A4 in R-SMCs. S100A4 is known to exert both extracellular and intracellular functions and has been found to translocate to the nucleus of human articular chondrocytes upon IL1-β stimulation to induce MMP-13 production^30^. This translocation required sumoylation of two lysine residues on S100A4 by the sumo-1 protein. Whether such a mechanism might be responsible for apelin nuclear localization remains to be determined.

To further explore the interplay between apelin and S100A4, we took advantage of our unique in vitro model, in which, by treating contractile and apelin-devoid S-SMCs with PDGF-BB, we promote the transition towards the synthetic R-SMCs. After PDGF-BB treatment, SMCs exhibited increased expression of apelin and S100A4, which corroborated our in vivo observations. The nuclear expression of apelin was positively correlated with S100A4 nuclear expression after PDGF-BB treatment or N. Ap overexpression. These results strongly suggest the existence of a new cell intrinsic function of apelin in SMCs. This pathway would allegedly target apelin to the cell nucleus where it might either facilitate S100A4 translocation or induce its transcription. This dual targeting would require an alternative translation start site (as shown in our experiments) that produces a protein isoform with a modified N-terminal sequence as described for other proteins such as VEGF, FGF2 and stress-activated protein kinase MK2^31,32^. Different mechanisms lead to the generation of protein isoforms from single mRNAs such as downstream AUG start codon usage or alternative non-AUG codon initiation, and these should be investigated to see if they might contribute to apelin’s nuclear localization. Furthermore, the mechanisms governing apelin/S100A4 interaction and sub-cellular localization are largely unknown. Xia et al.^33^ recently described a nuclear localization of S100A4 in mesenchymal progenitor cells responsible for their fibrogenicity. They showed that S100A4 was transported to the nucleus within a transportin1/S100A4/CD44 complex mediated by hyaluronan-dependent CD44 internalization. It has been previously shown that the binding of various apelin isoforms to their receptor induces APJ internalization and different sub-cellular targeting^11^. Nevertheless, whether apelin and S100A4 localization is dependent on receptor internalization and subsequent nuclear targeting in SMCs needs to be demonstrated. Of note, the S100A4 nuclear expression in mesenchymal progenitor cells has been shown to be transient whereas S100A4 is not found in the nucleus once the cells have initiated their fibrogenic transition^33^. This corroborates our findings that at higher levels of apelin, and once S-SMCs have initiated their phenotypic transition, S100A4 is excluded from the nucleus (Fig. 4C).

The phenotypic switch of SMCs induced by apelin was confirmed not only due to the change of cell morphology but also due to the typical increase of proliferation, decrease of α-SMA expression, and increased expression and release of S100A4. Other studies using mouse and rat SMCs under apelin-13 treatment are in line with our results, adding that the increase in proliferation takes place probably through the PI3K–Akt–ERK1/2–cyclin D1 and Jagged1–Notch3–cyclin D1 pathways^14,18,34^. Furthermore, the treatment of rat SMCs with apelin increases the levels of matrix metalloproteinase 2 (MMP2) and phosphorylation of Forkhead box O3a (FoxO3a)^19^, both increased in human atherosclerotic plaques^19^. We had previously shown that the promoter regions of the Egr-1 transcription factor, a key regulator of SMC phenotypic transition, are upregulated in porcine SMCs after PDGF-BB/oligomeric S100A4 stimulation^21.^ Liu et al.^35^ also reported an increase of Egr-1 expression during apelin-13-induced proliferation and migration of rat SMCs suggesting an overlap between the apelin and S100A4 pathways. Herein, we show that PDGF-BB stimulation of SMCs induces a direct interaction between apelin and S100A4, mediating a nuclear localization and a phenotypic switch towards dedifferentiated SMCs. Besides sumoylation and receptor-mediated internalization, we describe a possible new mechanism that leads to nuclear S100A4 through the interaction with apelin. Nevertheless, the kinetics and specificities of this interaction needs further investigation.

A close relationship between apelin and atherosclerosis has been demonstrated. It affects the behavior of the main actors of atherosclerosis (SMCs, ECs and macrophages), with some pathways being conserved between cell types. While in ECs and macrophages the results point to an anti-atherogenic anti-inflammatory role for apelin, it is still not clear how apelin affects SMCs. Reports have supported the pro-atherogenic role of apelin, showing that the intraperitoneal administration of apelin-13 in ApoE-/- mice exacerbated plaque burden in the aortic wall, through the AMPKα/PINK1 mitophagic pathway^36^. Additionally, the knockout of the APJ receptor in an ApoE-/- mouse model also showed a dramatic reduction of atherosclerotic lesions, probably due to a decrease of the reactive oxygen species (ROS) levels, without affecting the cholesterol content^14^. Other reports put forward the anti-atherosclerotic potential of apelin. Chun and colleagues showed that apelin decreases atherosclerosis progression, partly by increasing vascular NO expression, consequently decreasing ROS production^13^, while Fraga-Silva and collaborators^37^ show that apelin-13 intraperitoneal administration promoted the stabilization of atherosclerotic plaques, as well as the decrease of serum lipid content. The discrepancy between the different results might be attributed to the different models used, the impact of apelin on early atherosclerosis development versus late established plaque development, and the pleiotropic functions of the apelin/APJ system depending on cell type, tissue and disease. Nevertheless, our results clearly indicate that apelin promotes SMC dedifferentiation towards a synthetic phenotype characteristic of atherosclerosis development.

As previously described, several active forms of apelin exist with different functions. Unfortunately, within the limitations of our experiments, we were not able to discriminate between apelin isoforms, and further investigation is needed to describe which isoform is expressed in this model and is capable of translocating to the nucleus. Furthermore, establishing a definitive causal relationship between apelin and S100A4 in mediating SMC phenotypic changes requires additional investigation. We acknowledge these gaps in our current understanding and advocate for future studies to delve deeper into these aspects for a comprehensive view of the role of apelin and its relationship with S100A4 in SMC phenotypic changes.

In a nutshell, we show that apelin induces SMC phenotypic transition and acquisition of a dedifferentiated profile. We also unravel a potential new apelin pathway leading to the interaction and nuclear apelin and S100A4 localization. Thus, it will be important to decipher the complex relationship between apelin and S100A4, the mechanisms leading to apelin nuclear hijack and whether it might constitute a potential target in toning down SMC-driven atherosclerosis development.

## Materials and methods

### Animal specimens

All animal studies were performed after approval from institutional review board of the Swiss Federal Veterinary Office (“Autorité Cantonale, Direction Générale de la Santé”) and all methods were performed in accordance with the relevant guidelines and regulations (license numbers:31.1.1005/1720/II and 31.1.1005/1853/II). These animal studies conform to the guidelines from Directive 2010/63/EU of the European Parliament on the protection of animals used for scientific purposes. All animal experiments were performed in accordance with ARRIVE guidelines.

Three-month-old domestic crossbred pigs (*Sus scrofa*) were used. Intimal thickening was induced by direct self-expanding stent implantation (Wallstent, Schneider, Bulach, Switzerland) in the left anterior descending coronary artery ^38^. Injured coronary arteries were collected 30 days after stent implantation (n = 4). Non-stented right coronary arteries and left anterior descending coronary arteries of 8-month-old pigs obtained from a nearby slaughterhouse served as controls (n = 3). Intimal thickening was induced in adult rat (6 to 8 weeks old) thoracic aorta by removal of the endothelium using an inflated embolectomy catheter. Aortas were collected 20 days after injury (n = 5). Age-matched normal rat aortas as well as non-lesional area of a balloon-induced intimal thickening of rat aorta served as controls (n = 3). Tissue specimens were fixed with 10% neutral buffered formalin and embedded in paraffin (Miles Scientific, Naperville, IL).

### Cell culture and treatment

Coronary arteries of 8-month-old pigs were obtained from a nearby slaughterhouse. S-SMCs were isolated from the porcine coronary artery media using enzymatic digestion^20^. SMCs between the 6th and 11th passages were plated at a density of 60 cells/mm^2^ in 60 mm culture dishes containing Dulbecco’s modified eagle medium (DMEM, #10,566,016 Gibco-Invitrogen, Basel, Switzerland) supplemented with 10% fetal calf serum (FCS, Amimed, Bioconcept, Allschwil, Switzerland). After 24 h, S-SMCs were treated with 30 ng/ml of human recombinant PDGF-BB, (Fluka/Sigma-Aldrich, Saint-Louis, MI, USA) for 96 h to induce phenotypic changes and/or S100A4 upregulation.

### Proximity ligation assay

S-SMCs were treated with 30 ng/ml of human recombinant PDGF-BB, (Fluka/Sigma-Aldrich, Saint-Louis, MI, USA) to induce phenotypic changes. After 96 h of stimulation, cells were fixed in 1% paraformaldehyde (PFA, Fluka/Sigma-Aldrich) washed in PBS and permeabilized in 0.1% triton X-100 (Fluka/Sigma-Aldrich). Proximity ligation assay (PLA) was performed using reagents provided in a NaveniFlex 100 Mouse/Rabbit kit (Navinci Diagnostics, NF.MR.100) according to the manufacturer’s instructions. After blocking non-specific binding, cells were incubated overnight at 4 °C with primary antibodies. Antibodies used were rabbit polyclonal anti-apelin antibody (PA5-114860, Invitrogen) and mouse monoclonal anti-S100A4 antibody (AMAB90596, Sigma-Aldrich). Secondary antibody incubation, ligation and amplification reactions as well as DAPI counterstaining were performed as per the manufacturer’s instructions. Atto 488 detection fluorophore was used. Non-stimulated S-SMCs were used as basal level signal and omitting to add one of the primary antibodies was used as negative control. Slides were mounted in buffered polyvinyl alcohol. Images were taken by means of an Axioskop 2 microscope (Carl Zeiss, Jena, Germany) equipped with a plan-neofluar × 20/0.50 objective and a high sensitivity, high resolution digital colour camera (Axiocam, Carl Zeiss) using the software Zen 2.3 (Carl Zeiss) and processed using Adobe Photoshop. Quantification of PLA complexes/cell was done using the QuPath image analysis software version 0.4.3.

### Generation of nuclear and secreted preproapelin

**Figure 5:**
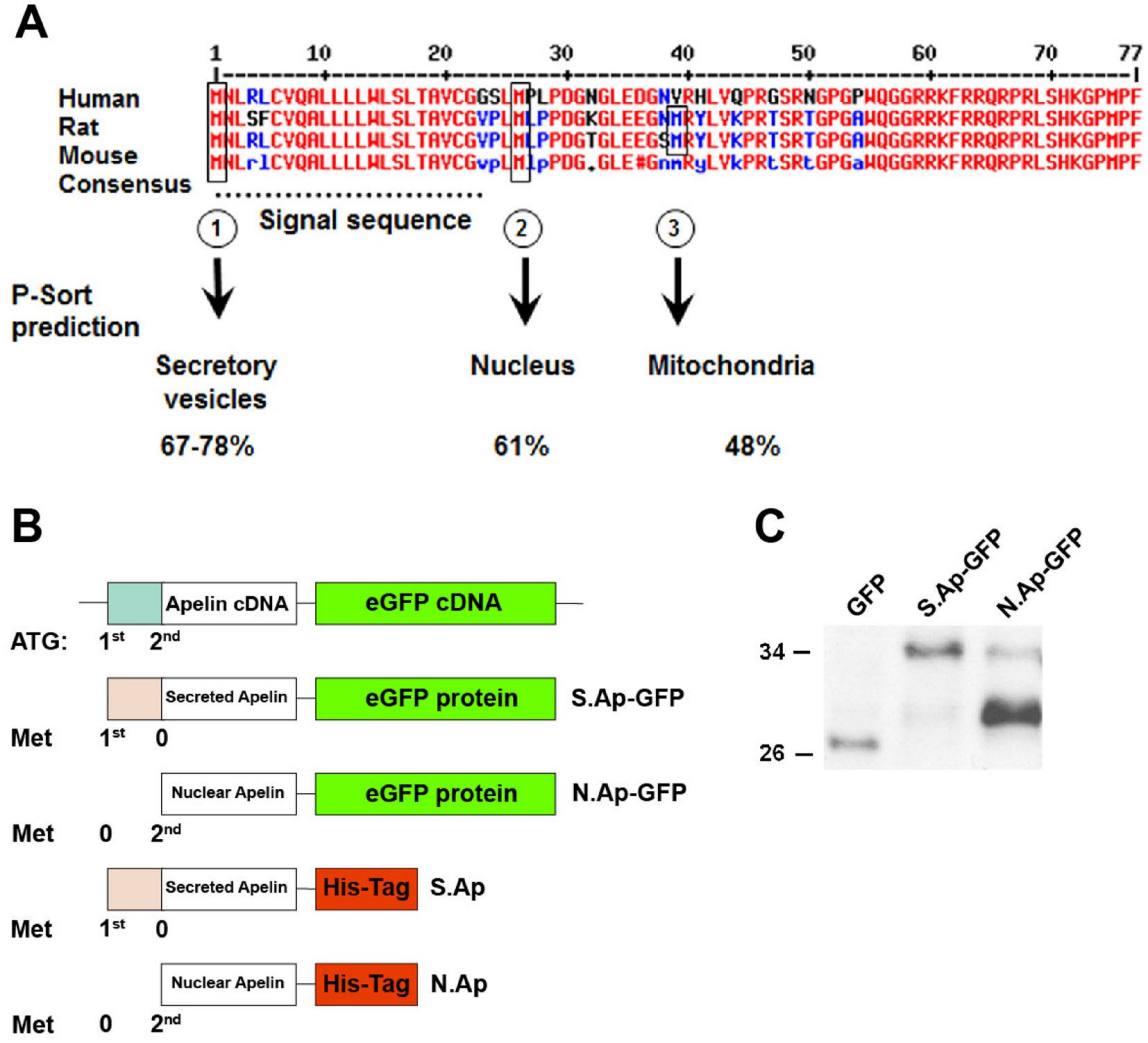
Apelin translation at first or second initiation site. (**A**) Alignment of human, rat, and mouse preproApelin amino acid sequences and consensus sequence showing initiation sites at position 1 (①), 26 (②) and 39 (③). P-sort prediction for translation initiation starting at the first, second and third initiation site leads to signal sequences targeting proapelin to secretory vesicles, and shorter proapelin fragments to the nucleus and mitochondria, respectively. (**B**) Scheme showing point mutations generated at the first or second ATG site to abolish translation initiation with eGFP or His-Tagged apelin. (**C**) Western blot analysis of protein extracts 24 h after transfection of empty pEGFP-N1 (GFP), both mutated preproapelin encoded pEGFP-N1 (S. Ap-GFP and N. Ap-GFP) plasmids that were detected with GFP. GFP and fusion proteins are detected at the expected molecular weight. The figure represents a cropped image retaining important bands, original blots are presented in page 13 of the supplementary file.

The alignment of preproapelin amino acid sequences derived from human, rat, and mouse preproapelin showed 2 translation initiation sites in human, and 3 in rat and mouse (Fig. 5A). As previously published for 18 secretory hormone and neuropeptide precursors^39^, P-sort analysis was applied to preproapelin, and predicted different subcellular localizations depending on each ATG translation initiation site. The first initiation site showed the highest probability (67–78%) and led to the presence of a signal sequence and thus targeting of preproapelin to secretory vesicles. The second initiation site obtained 61% of probability and resulted in nuclear localization. The third initiation site, present only in mouse and rat sequences, with 48% probability, led to mitochondrial destination. The first or second ATG sites, coding for methionine, were mutated to TTG by site-specific directed mutagenesis PCR (Fig. 5B). Mutation of the first ATG disrupted the full translation of the peptide, leading to N terminal-signal peptide-truncated apelin, which was mainly targeted to the nucleus. Mutation of the second ATG gave rise to wild type preproapelin and apelin was mainly directed towards the secretory vesicles. Both mutated preproapelin were cloned into pEGFP-N1 (N. Ap-GFP and S. Ap-GFP) vector or into His-tag expressing pcDNA3.1 vector (N. Ap and S. Ap).

Expression of mutated preproapelin/eGFP fusion proteins 24 h after transfection was assessed by Western blot using GFP antibody (Fig. 5C). After transfection of empty pEGFP-N1 plasmid, eGFP was detected at 27 kDa, corresponding to the molecular weight of eGFP. Mutated preproapelin/eGFP fusion protein was detected at 37 kDa for S. Ap-GFP and 32 kDa for N. Ap-GFP at the expected molecular weight (Fig. 5C). A faint signal for S. Ap-GFP fusion protein was observed in the N. Ap-GFP sample and likely corresponds to a minor non-ATG-initiated translation of S. Ap-GFP.

### Transfection of mutated preproapelin-EGFP or His-tag and human S100A4 encoded plasmids

Mutated preproapelin encoded pEGFP-N1 or pcDNA3.1 plasmids and empty pEGFP-N1 or empty pcDNA3.1 plasmids as negative controls were used. Transfection of plasmids (2 µg/mL) was performed on adherent SMCs using Lipofectamine 2000 (2 µl/mL, Gibco-Invitrogen) in OptiMEM (Gibco-Invitrogen). After 6 h, the medium was replaced with DMEM containing 10% FCS for 4 days. S100A4 transfected SMC supernatants were collected after 48 h for competitive ELISA assay. After 4 days, cells were fixed and processed for immunofluorescence staining or harvested for Western blotting. These experiments were repeated at least 3 times for each transfection.

### Immunofluorescence staining and Immunohistochemistry

Double immunofluorescence staining was performed on adherent SMCs. Cells were fixed for 30 min in DMEM with 2% HEPES (Gibco-Invitrogen) and 1% paraformaldehyde (PFA, Fluka/Sigma-Aldrich), then rinsed in PBS and further incubated for 5 min in methanol at -20 °C. After washing in PBS, cells were double stained with mouse monoclonal IgM antibody recognizing the C-terminal sequence of S100A4^22^ or with rabbit polyclonal IgG specific apelin (raised against human/rodent apelin-13, and provided by Dr Audigier)^40^. Rabbit polyclonal anti-apelin (FL-77) antibody was purchased from Santa Cruz (#sc-33804)^41^ and was also used for immunohistochemistry and immunofluorescence staining on porcine and human tissues. Alexa 594-conjugated goat anti-mouse IgM and Alexa-488-conjugated goat anti-rabbit IgG were used as secondary antibodies (Molecular Probes, Eugene, OR, USA). Nuclei were stained with 4',6-diamidino-2-phenylindole dihydrochloride (DAPI, Fluka/Sigma-Aldrich). Slides were mounted in buffered polyvinyl alcohol. Images were taken by means of an Axioskop 2 microscope (Carl Zeiss, Jena, Germany) equipped with a plan-neofluar × 20/0.50 objective and a high sensitivity, high resolution digital colour camera (Axiocam, Carl Zeiss) using the software Zen 2.3 (Carl Zeiss) and processed using Adobe Photoshop.

Immunohistochemical staining for apelin was performed on 4 μm-thick paraffin sections of porcine and rat coronary artery specimens. Immunoreactivity was intensified by microwave treatment (750 W, 5 min) in citrate buffer (10 mM, pH 6.0). Biotinylated-goat anti-mouse antibody was used as secondary antibody (Dako). The presence of the specific protein was revealed by means of the streptavidin–biotin peroxidase complex method (Dako). Slides were counterstained with hemalun and mounted in Eukitt (Kindler, Freiburg, Germany). Images were taken by means of an Axioskop microscope (Carl Zeiss, Jena, Germany) equipped with Plan-Neofluar × 20/0.3 objective and a high sensitivity, high resolution digital color camera (Axiocam, Carl Zeiss).

Double immunofluorescence staining on paraffin sections was also performed^42^. Before using the first antibodies, immunoreactivity was intensified by microwave treatment (250W, 20 min) in Tris/ethylenediaminetetraacetic acid (Tris/EDTA, 10 mM/1 mM, pH 9.1). Specimens were then stained with anti-apelin and anti-α-SMA. Alexa 488-conjugated goat anti-rabbit and Alexa 568-conjugated goat anti-mouse IgG2a were used as secondary antibodies, respectively (Molecular probes). Nuclei were stained with DAPI.

### Confocal image acquisition

For quantification of double immunofluorescence staining in fixed SMCs, a Nikkon Diaphot 300 inverted microscope with a 100 × oil immersion objective (NA 1.3), equipped with a Nipkoff confocal spinning wheel (Visitech), a CoolSnap HQ cooled CCD camera (Visitron Systems), 488 nm and 568 nm Laser illumination with an electronically controlled wavelength selector (Visitech9, and Universal Imaging Metamorph software) was used. Quantification of nuclear (NUC) versus cytoplasmic (CYTO) apelin and S100A4 in S-SMCs treated with PDGF-BB vs control (Ctl) using charge-couple device (CCD) photon detector for each wavelength channel. Fluorochrome intensity is given in CCD units/5 s. The same method was used to quantify nuclear apelin and nuclear S100A4 after N.Ap or S.Ap plasmid transfection. ∆CCD corresponds to the total intensity minus background.

Double immunofluorescence staining was acquired using a confocal laser scan microscope (LSM700, Carl Zeiss) through an oil immersion Plan-Neofluar 40x/1.30 oil objective. Contrast and color adjustment of pictures, when required, were done using Zen software (Carl Zeiss) and applied across the integrality of the images.

### Cell proliferation

Transfected SMCs were synchronised in serum-free medium overnight^43^, incubated with 5-bromo-2'-deoxyuridine (BrdU, 10^−5^ M, Fluka/Sigma-Aldrich) for 18 h at 37 °C and then fixed for 5 min in methanol at  − 20 °C. After washing in PBS they were incubated for 20 min in 1 M HCl followed by 0.1 M Borax, pH 8.5 for 5 min. The incorporated BrdU was detected immunohistochemically using a mouse monoclonal BrdU antibody (Dako). Alexa 488-conjugated goat anti-mouse IgG (Molecular Probes) was used as secondary antibody. Nuclei were stained with DAPI. Slides were mounted in buffered polyvinyl alcohol. The percentage of BrdU-positive cells was calculated using Metamorph 6.0 image analysis system (Universal Imaging Corporation, Biocompare, San Francisco, CA).

### Western blots

Proteins were extracted from cultured SMCs as previously described^22,23^. Protein concentration was determined according to Bradford^44^. Proteins were separated by SDS-PAGE on 4–12% minigels (Bio-Rad, Basel, Switzerland) and stained with Coomassie brilliant blue (R250, Fluka). For Western blotting 1 to 12 µg of protein were electrophoresed, transferred to a nitrocellulose membrane (Protran® 0.2 µm; Schleicher and Schuell, Dassel, Germany) for α-SMA or PVDF membrane (0.45 µm, ImmobilonTM-P, Millipore Corporation, Bedford, MA) for S100A4^22^. Vimentin was used as a housekeeping protein, and detected using a specific mouse monoclonal IgG1 anti-vimentin (clone V9, DAKO). Membranes were cut horizontally between 25 and 35 kDa and incubated with anti-α-SMA or anti-vimentin (upper part of the membrane) or anti-S100A4 (mouse monoclonal IgG1 anti-S100A4 antibody AMAB90596, Sigma-Aldrich or homemade mouse monoclonal IgM antibody recognizing the C-terminal sequence of S100A4^22^, lower part of the membrane) which explains the lack of full-length blots in some of the experiments (supplementary file page 7–9). All corresponding samples (α-SMA, vimentin or S100A4) derive from the same experiment and were processed in parallel. Horseradish peroxidase-conjugated goat anti-mouse IgG or IgM or goat anti-rabbit IgG were used as secondary antibodies. Enhanced chemiluminescence was used for detection (Amersham, Buckinghamshire, United Kingdom). Signals were digitized by means of Epson perfection 4990 photo scanner and analysed using Metamorph 6.0 image analysis system (Universal Imaging Corporation).

### Enzyme-linked immunosorbent assay (ELISA)

Supernatants from SMCs transfected with empty or mutated preproapelin-His-tag encoded plasmids (N. Ap or S. Ap) were collected 48 h after plating for competitive ELISA assay as previously described^23^. Briefly, mouse monoclonal S100A4 IgM and SMC supernatants were mixed for 1 h at room temperature and then incubated overnight at 4 °C in a 96 well-plate coated with the C-terminal 16 amino acids of S100A4 bound to BSA (20 ng/mL in 50 mM sodium bicarbonate, pH 8). Incubation with alkaline phosphatase-conjugated goat anti-mouse IgM (Southern Biotech) diluted in DMEM containing 10% FCS was performed 1 h at 37 °C. The substrate solution (p-nitrophenylphosphate; S0942, Fluka/Sigma-Aldrich) was added and the enzymatic reaction produced a soluble yellow product measured at 405 nm. The standard curve was performed by incubating the mouse monoclonal anti-S100A4 with increasing concentrations of S100A4 peptide instead of SMC supernatants.

### Statistical analysis

Results are shown as mean ± SEM. Quantification of nuclear apelin and nuclear S100A4 was performed using a Student’s t-test. Comparisons between treated and control groups were analyzed by one-way ANOVA or Kruskal–Wallis non-parametric ANOVA. Quantification of nuclear and cytoplasmic apelin and S100A4 was done by generating R squared and p values using Pearson’s correlation, slope values were generated by performing a simple linear regression. PLA quantification analysis was done with a Mann–Whitney non-parametric test. All statistical analysis was done using the Graphpad Prism v9.5 software. Differences were considered statistically significant at values of *P* < 0.05.

### Supplementary Information

Supplementary Information.

## Data Availability

All data generated or analysed during this study are included in this published article [and its supplementary information files].

## References

[1] Tatemoto K, Hosoya M, Habata Y, Fujii R, Kakegawa T, Zou MX, Kawamata Y, Fukusumi S, Hinuma S, Kitada C, Kurokawa T, Onda H, Fujino M (1998). Isolation and characterization of a novel endogenous peptide ligand for the human APJ receptor. Biochem. Biophys. Res. Commun..

[2] Kleinz MJ, Davenport AP (2005). Emerging roles of apelin in biology and medicine. Pharmacol. Ther..

[3] Japp AG, Newby DE (2008). The apelin-APJ system in heart failure: Pathophysiologic relevance and therapeutic potential. Biochem. Pharmacol..

[4] Pitkin SL, Maguire JJ, Kuc RE, Davenport AP (2010). Modulation of the apelin/APJ system in heart failure and atherosclerosis in man. Br. J. Pharmacol..

[5] Zhong JC, Yu XY, Huang Y, Yung LM, Lau CW, Lin SG (2007). Apelin modulates aortic vascular tone via endothelial nitric oxide synthase phosphorylation pathway in diabetic mice. Cardiovasc. Res..

[6] Novakova, V., Sandhu, G. S., Dragomir-Daescu, D., & Klabusay, M. Apelinergic system in endothelial cells and its role in angiogenesis in myocardial ischemia. *Vascul. Pharmacol.* (2015). 10.1016/j.vph.2015.08.005 26254105

[7] Zhong JC, Zhang ZZ, Wang W, McKinnie SMK, Vederas JC, Oudit GY (2017). Targeting the apelin pathway as a novel therapeutic approach for cardiovascular diseases. Biochim. Biophys. Acta Mol. Basis Dis..

[8] Maguire JJ, Kleinz MJ, Pitkin SL, Davenport AP (2009). [Pyr1]apelin-13 identified as the predominant apelin isoform in the human heart: vasoactive mechanisms and inotropic action in disease. Hypertension.

[9] Azizi M, Iturrioz X, Blanchard A, Peyrard S, De Mota N, Chartrel N, Vaudry H, Corvol P, Llorens-Cortes C (2008). Reciprocal regulation of plasma apelin and vasopressin by osmotic stimuli. J. Am. Soc. Nephrol..

[10] Shin K, Pandey A, Liu XQ, Anini Y, Rainey JK (2013). Preferential apelin-13 production by the proprotein convertase PCSK3 is implicated in obesity. FEBS Open Bio..

[11] Chaves-Almagro C, Castan-Laurell I, Dray C, Knauf C, Valet P, Masri B (2015). Apelin receptors: From signaling to antidiabetic strategy. Eur. J. Pharmacol..

[12] Wang W, McKinnie SM, Farhan M, Paul M, McDonald T, McLean B, Llorens-Cortes C, Hazra S, Murray AG, Vederas JC, Oudit GY (2016). Angiotensin-converting enzyme 2 metabolizes and partially inactivates pyr-apelin-13 and apelin-17: Physiological effects in the cardiovascular system. Hypertension.

[13] Chun HJ, Ali ZA, Kojima Y, Kundu RK, Sheikh AY, Agrawal R, Zheng L, Leeper NJ, Pearl NE, Patterson AJ, Anderson JP, Tsao PS, Lenardo MJ, Ashley EA, Quertermous T (2008). Apelin signaling antagonizes Ang II effects in mouse models of atherosclerosis. J. Clin. Invest..

[14] Hashimoto T, Kihara M, Imai N, Yoshida S, Shimoyamada H, Yasuzaki H, Ishida J, Toya Y, Kiuchi Y, Hirawa N, Tamura K, Yazawa T, Kitamura H, Fukamizu A, Umemura S (2007). Requirement of apelin-apelin receptor system for oxidative stress-linked atherosclerosis. Am. J. Pathol..

[15] Kojima Y, Kundu RK, Cox CM, Leeper NJ, Anderson JA, Chun HJ, Ali ZA, Ashley EA, Krieg PA, Quertermous T (2010). Upregulation of the apelin-APJ pathway promotes neointima formation in the carotid ligation model in mouse. Cardiovasc. Res..

[16] Zhou Y, Wang Y, Qiao S (2014). Apelin: a potential marker of coronary artery stenosis and atherosclerotic plaque stability in ACS patients. Int. Heart J..

[17] Tasci I, Dogru T, Naharci I, Erdem G, Yilmaz MI, Sonmez A, Bingol N, Kilic S, Bingol S, Erikci S (2007). Plasma apelin is lower in patients with elevated LDL-cholesterol. Exp. Clin. Endocrinol. Diabetes.

[18] Li L, Li L, Xie F, Zhang Z, Guo Y, Tang G, Lv D, Lu Q, Chen L, Li J (2013). Jagged-1/Notch3 signaling transduction pathway is involved in apelin-13-induced vascular smooth muscle cells proliferation. Acta Biochim. Biophys. Sin (Shanghai).

[19] Wang C, Wen J, Zhou Y, Li L, Cui X, Wang J, Pan L, Ye Z, Liu P, Wu L (2015). Apelin induces vascular smooth muscle cells migration via a PI3K/Akt/FoxO3a/MMP-2 pathway. Int. J. Biochem. Cell Biol..

[20] Hao H, Ropraz P, Verin V, Camenzind E, Geinoz A, Pepper MS, Gabbiani G, Bochaton-Piallat ML (2002). Heterogeneity of smooth muscle cell populations cultured from pig coronary artery. Arterioscler. Thromb. Vasc. Biol..

[21] Sakic A, Chaabane C, Ambartsumian N, Klingelhofer J, Lemeille S, Kwak BR, Grigorian M, Bochaton-Piallat ML (2022). Neutralization of S100A4 induces stabilization of atherosclerotic plaques: Role of smooth muscle cells. Cardiovasc. Res..

[22] Brisset AC, Hao H, Camenzind E, Bacchetta M, Geinoz A, Sanchez JC, Chaponnier C, Gabbiani G, Bochaton-Piallat ML (2007). Intimal smooth muscle cells of porcine and human coronary artery express S100A4, a marker of the rhomboid phenotype in vitro. Circ. Res..

[23] Chaabane C, Heizmann CW, Bochaton-Piallat ML (2015). Extracellular S100A4 induces smooth muscle cell phenotypic transition mediated by RAGE. Biochim. Biophys. Acta..

[24] Allahverdian S, Chaabane C, Boukais K, Francis GA, Bochaton-Piallat ML (2018). Smooth muscle cell fate and plasticity in atherosclerosis. Cardiovasc. Res..

[25] Bennett MR, Sinha S, Owens GK (2016). Vascular smooth muscle cells in atherosclerosis. Circ. Res..

[26] Grootaert MOJ, Bennett MR (2021). Vascular smooth muscle cells in atherosclerosis: Time for a re-assessment. Cardiovasc. Res..

[27] Liu M, Gomez D (2019). Smooth muscle cell phenotypic diversity. Arterioscler. Thromb. Vasc. Biol..

[28] Shankman LS, Gomez D, Cherepanova OA, Salmon M, Alencar GF, Haskins RM, Swiatlowska P, Newman AA, Greene ES, Straub AC, Isakson B, Randolph GJ, Owens GK (2015). KLF4-dependent phenotypic modulation of smooth muscle cells has a key role in atherosclerotic plaque pathogenesis. Nat. Med..

[29] Alencar GF, Owsiany KM, Karnewar S, Sukhavasi K, Mocci G, Nguyen AT, Williams CM, Shamsuzzaman S, Mokry M, Henderson CA, Haskins R, Baylis RA, Finn AV, McNamara CA, Zunder ER, Venkata V, Pasterkamp G, Bjorkegren J, Bekiranov S, Owens GK (2020). Stem cell pluripotency genes Klf4 and Oct4 regulate complex SMC phenotypic changes critical in late-stage atherosclerotic lesion pathogenesis. Circulation.

[30] Miranda KJ, Loeser RF, Yammani RR (2010). Sumoylation and nuclear translocation of S100A4 regulate IL-1beta-mediated production of matrix metalloproteinase-13. J. Biol. Chem..

[31] Touriol C, Bornes S, Bonnal S, Audigier S, Prats H, Prats AC, Vagner S (2003). Generation of protein isoform diversity by alternative initiation of translation at non-AUG codons. Biol. Cell..

[32] Trulley P, Snieckute G, Bekker-Jensen D, Menon MB, Freund R, Kotlyarov A, Olsen JV, Diaz-Munoz MD, Turner M, Bekker-Jensen S, Gaestel M, Tiedje C (2019). Alternative translation initiation generates a functionally distinct isoform of the stress-activated protein kinase MK2. Cell Rep..

[33] Xia H, Herrera J, Smith K, Yang L, Gilbertsen A, Benyumov A, Racila E, Bitterman PB, Henke CA (2021). Hyaluronan/CD44 axis regulates S100A4-mediated mesenchymal progenitor cell fibrogenicity in idiopathic pulmonary fibrosis. Am. J. Physiol. Lung Cell Mol. Physiol..

[34] Liu C, Su T, Li F, Li L, Qin X, Pan W, Feng F, Chen F, Liao D, Chen L (2010). PI3K/Akt signaling transduction pathway is involved in rat vascular smooth muscle cell proliferation induced by apelin-13. Acta Biochim. Biophys. Sin (Shanghai).

[35] Liu QF, Yu HW, You L, Liu MX, Li KY, Tao GZ (2013). Apelin-13-induced proliferation and migration induced of rat vascular smooth muscle cells is mediated by the upregulation of Egr-1. Biochem. Biophys. Res. Commun..

[36] He L, Zhou Q, Huang Z, Xu J, Zhou H, Lv D, Lu L, Huang S, Tang M, Zhong J, Chen JX, Luo X, Li L, Chen L (2019). PINK1/Parkin-mediated mitophagy promotes apelin-13-induced vascular smooth muscle cell proliferation by AMPKalpha and exacerbates atherosclerotic lesions. J. Cell Physiol..

[37] Fraga-Silva RA, Seeman H, Montecucco F, da Silva AR, Burger F, Costa-Fraga FP, Anguenot L, Mach F, Dos Santos RAS, Stergiopulos N, da Silva RF (2018). Apelin-13 treatment enhances the stability of atherosclerotic plaques. Eur. J. Clin. Invest..

[38] Christen T, Verin V, Bochaton-Piallat M, Popowski Y, Ramaekers F, Debruyne P, Camenzind E, van Eys G, Gabbiani G (2001). Mechanisms of neointima formation and remodeling in the porcine coronary artery. Circulation.

[39] Brun C, Philip-Couderc P, Raggenbass M, Roatti A, Baertschi AJ (2006). Intracellular targeting of truncated secretory peptides in the mammalian heart and brain. FASEB J..

[40] Boucher J, Masri B, Daviaud D, Gesta S, Guigne C, Mazzucotelli A, Castan-Laurell I, Tack I, Knibiehler B, Carpene C, Audigier Y, Saulnier-Blache JS, Valet P (2005). Apelin, a newly identified adipokine up-regulated by insulin and obesity. Endocrinology.

[41] Picault FX, Chaves-Almagro C, Projetti F, Prats H, Masri B, Audigier Y (2014). Tumour co-expression of apelin and its receptor is the basis of an autocrine loop involved in the growth of colon adenocarcinomas. Eur. J. Cancer.

[42] Coen M, Burkhardt K, Bijlenga P, Gabbiani G, Schaller K, Kovari E, Rufenacht DA, Ruiz DS, Pizzolato G, Bochaton-Piallat ML (2013). Smooth muscle cells of human intracranial aneurysms assume phenotypic features similar to those of the atherosclerotic plaque. Cardiovasc. Pathol..

[43] Stouffer GA, Owens GK (1992). Angiotensin II-induced mitogenesis of spontaneously hypertensive rat-derived cultured smooth muscle cells is dependent on autocrine production of transforming growth factor-b. Circ. Res..

[44] Bradford MM (1976). A rapid and sensitive method for the quantification of microgram quantities of protein utilizing the principle of protein-dye binding. Anal. Biochem..

