# Supplementary material for: Apelin is expressed in intimal smooth muscle cells and promotes their phenotypic transition
Source: Sci Rep. 2023 Oct 31;13:18736. doi: 10.1038/s41598-023-45470-z (PMC10618247; doi:10.1038/s41598-023-45470-z)
Supplement: Supplementary file 1 — Supplementary Information. [file 41598_2023_45470_MOESM1_ESM.pdf]

# Apelin is expressed in intimal smooth muscle cells and promotes their phenotypic transition

***Supplementary raw data***

**Luís Miguel Cardoso Dos Santos[1]*, Pascal Azar[1]*, Cécile Brun[2], Stéphane König[3], Angela Roatti[4], Alex J Baertschi[4], Chiraz Chaabane[1], Marie-Luce Bochaton-Piallat[1]**

[1]Department of Pathology and Immunology, [2]Geneva University Hospitals, [3]Department of Neuroscience and [4]Department of Physiology and metabolism, Faculty of Medicine, University of Geneva Switzerland.

*: Both authors contributed equally to this work.

**Short title**: Apelin and smooth muscle cells

**Corresponding author**: Prof. Marie-Luce Bochaton-Piallat

University of Geneva-CMU

Department of Pathology and Immunology

Rue Michel Servet -1

1211 Geneva 4, Switzerland

Tel: +41-22-379-5764, Fax: +41-22-379-5746

Email: Marie-Luce.Piallat@unige.ch

**Figure 1A**

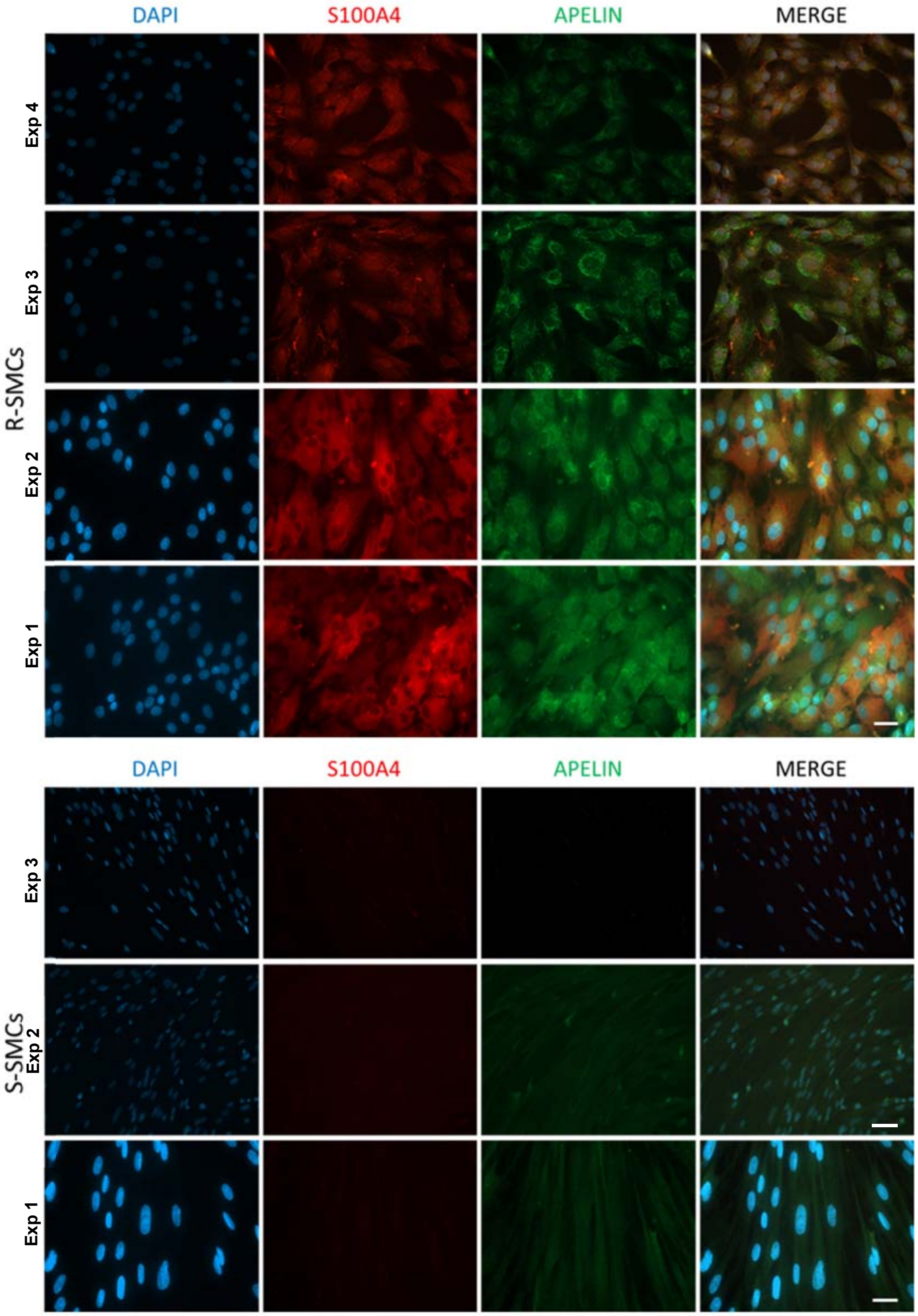


*Double immunofluorescence staining showing apelin and S100A4 expression in R-SMCs and S-SMCs from 4 and 3 independent cell cultures respectively, nuclei are stained in blue with DAPI. Scale bar for R-SMCs and S-SMCs Exp 1 30µm, for S-SMCs Exp 2 and Exp 3 60µm.*

**Figure 1B**

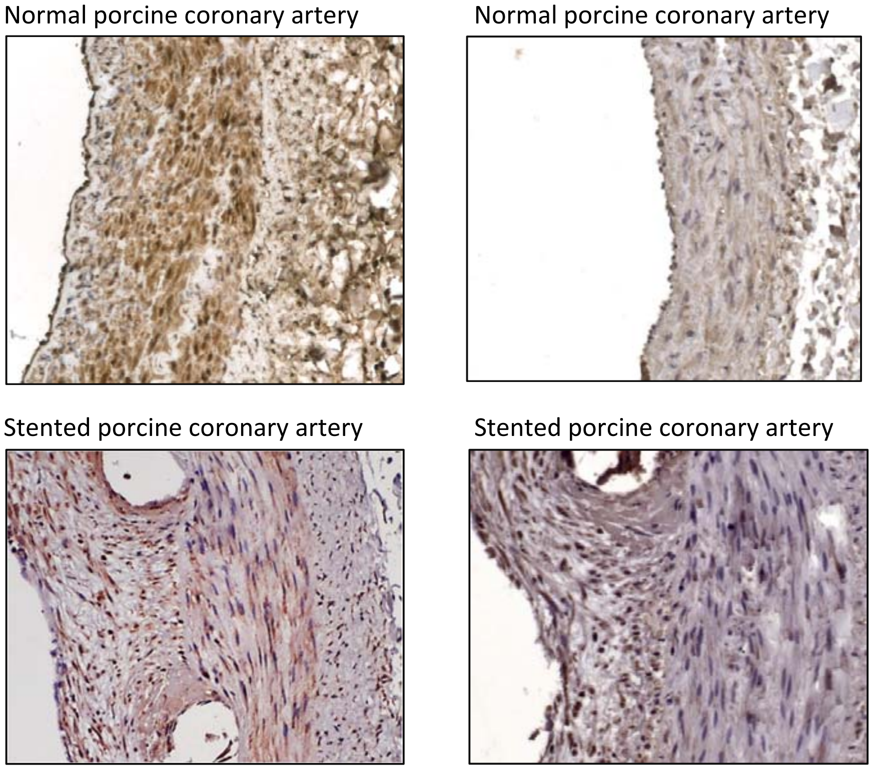


*Immunohistochemistry showing expression of apelin in normal and stented porcine coronary arteries. N=2 for each condition*

**Figure 1 D**

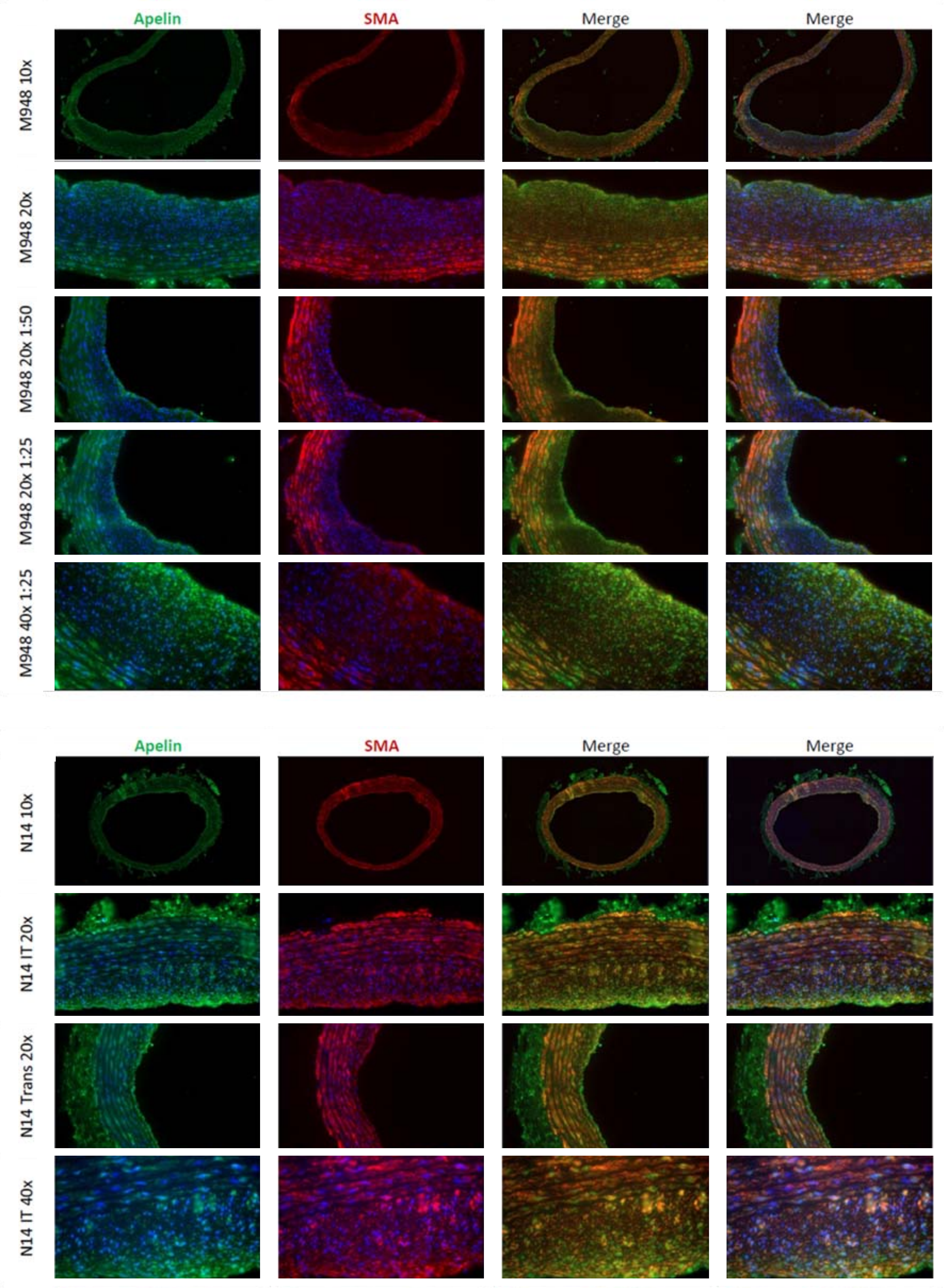
Apelin
SMA
Merge
Merge
M948 10x
M948 20x
M948 20x 1:50
M948 20x 1:25
M948 40x 1:25
Apelin
SMA
Merge
Merge
N14 10x
N14 IT 20x
N14 Trans 20x
N14 IT 40x

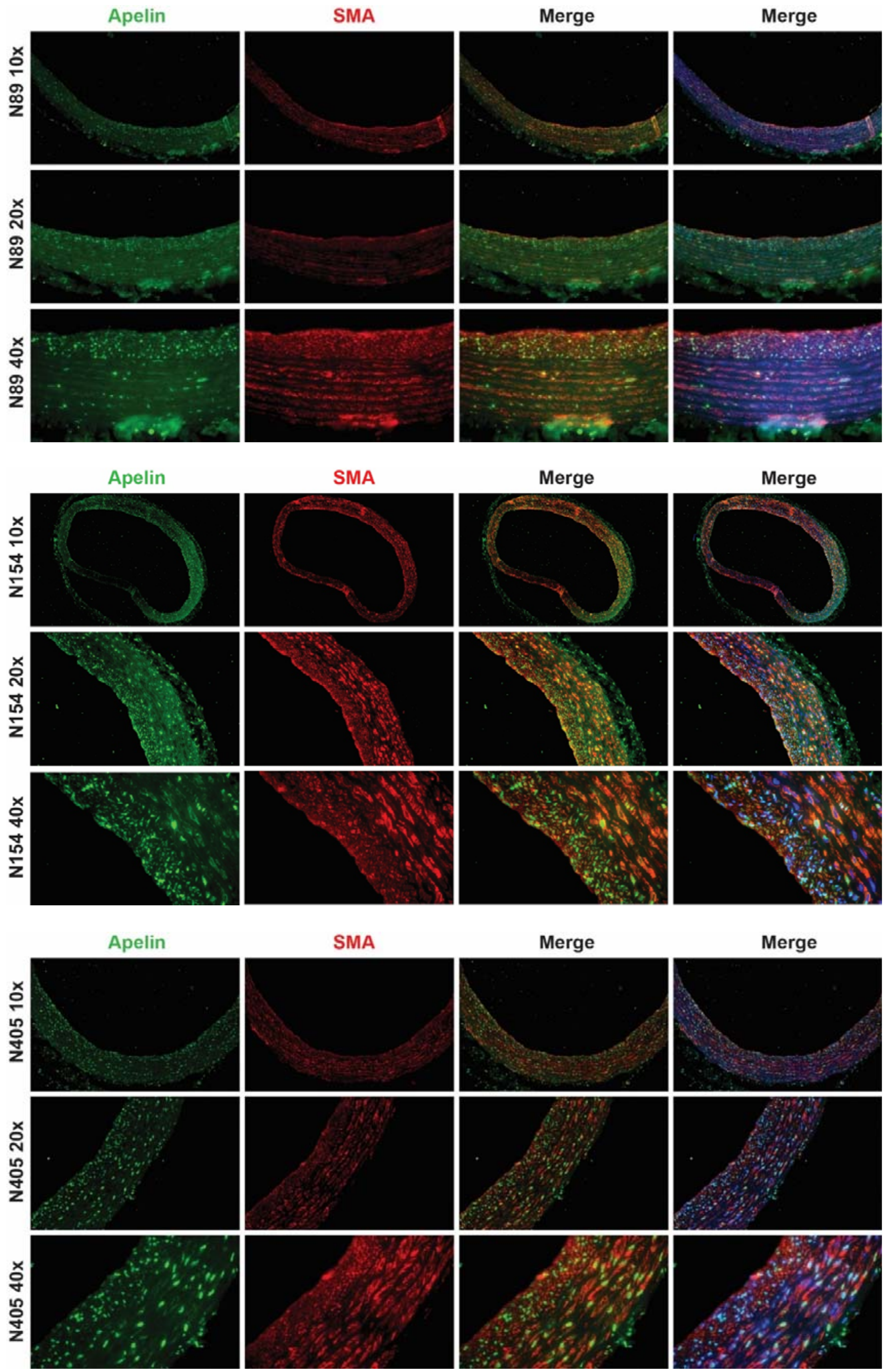
Apelin
SMA
Merge
Merge
N89 10x
N89 20x
N89 40x
Apelin
SMA
Merge
Merge
N154 10x
N154 20x
N154 40x
Apelin
SMA
Merge
Merge
N405 10x
N405 20x
N405 40x

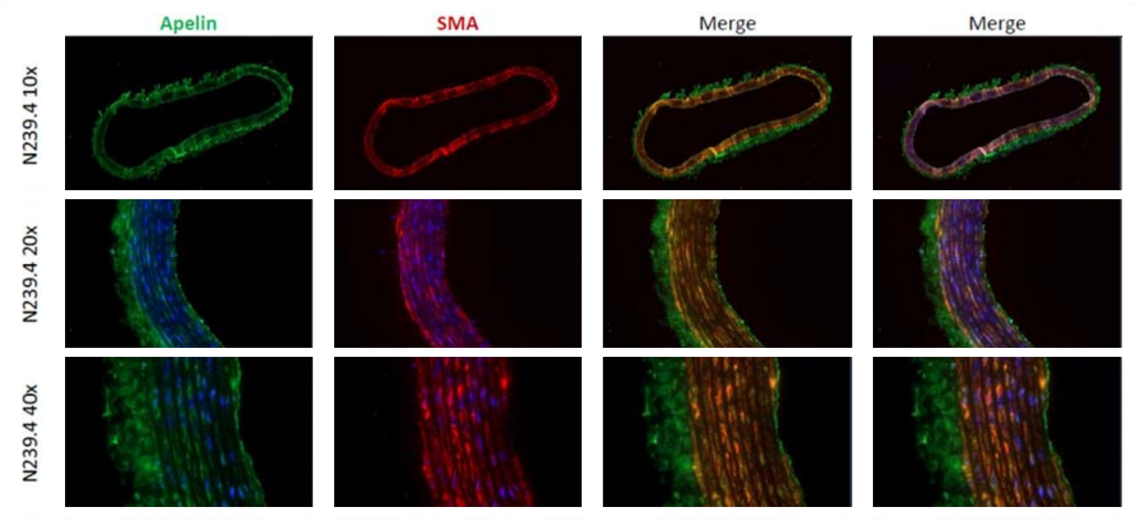


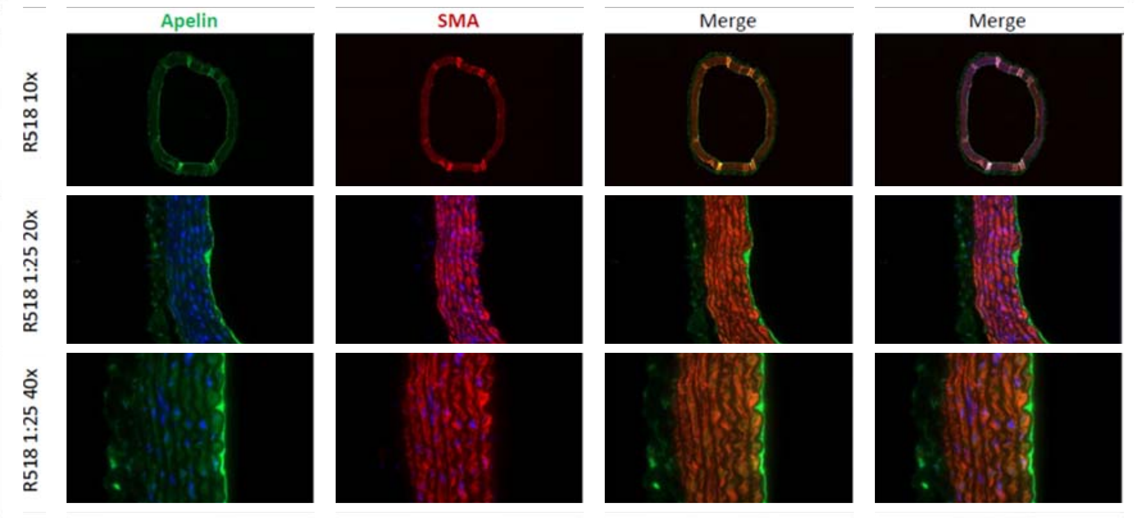


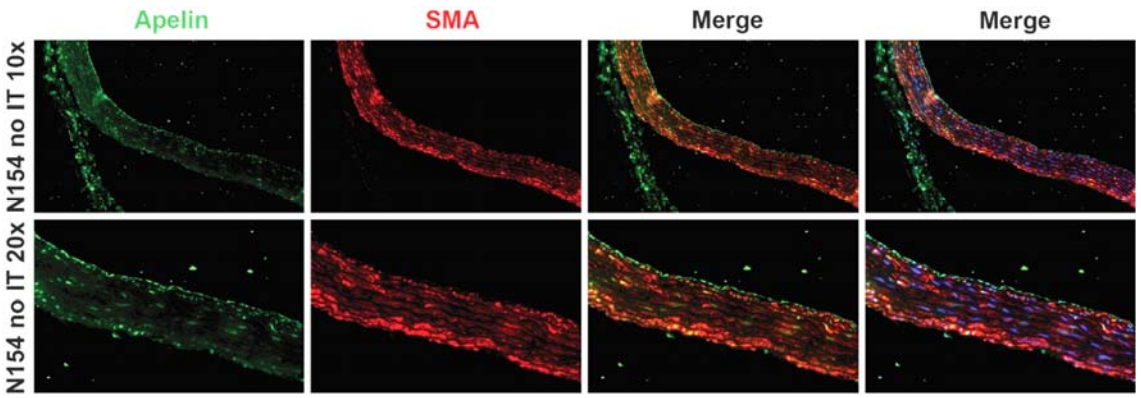


*Double immunofluorescence staining showing expression of α-SMA (red) and apelin (green) in normal and balloon-induced intimal thickening of rat aorta. M914, N14, N89, N154 and N405 are samples from 20 days induced intimal thickening of rat aortas and N239.4 and R518 are normal rat aortas. N154 no IT (no intimal thickening) sample represents a non-lesional area of the N154 rat aorta used as a control. 1:25 and 1:50 represent the antibody dilutions used.*

**Figure 3B**

S100A4 and α-SMA Western Blots:

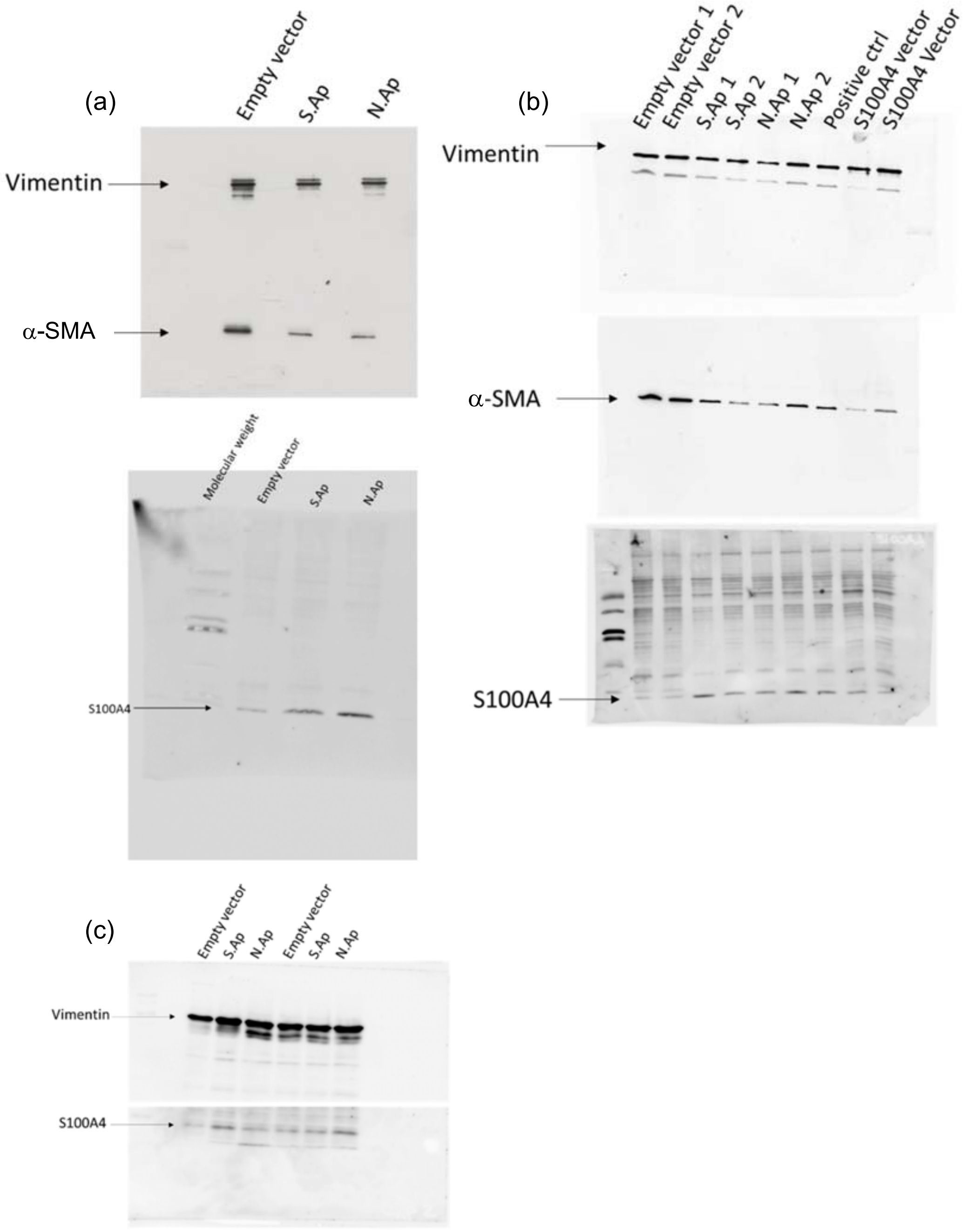

(d)

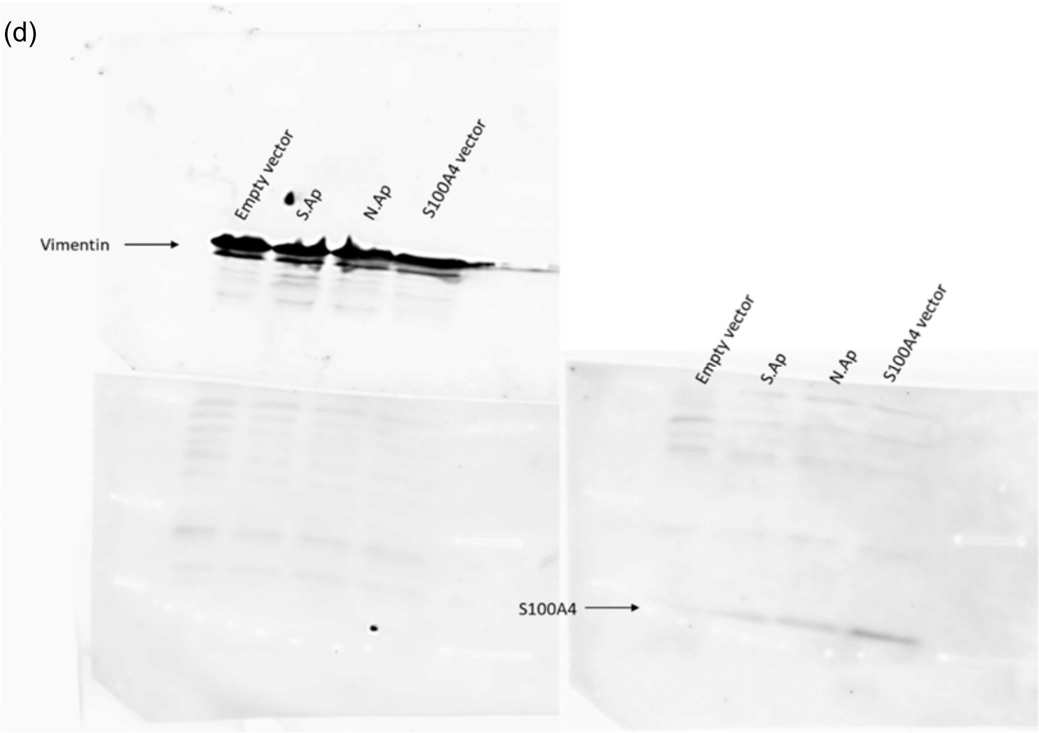


(e)

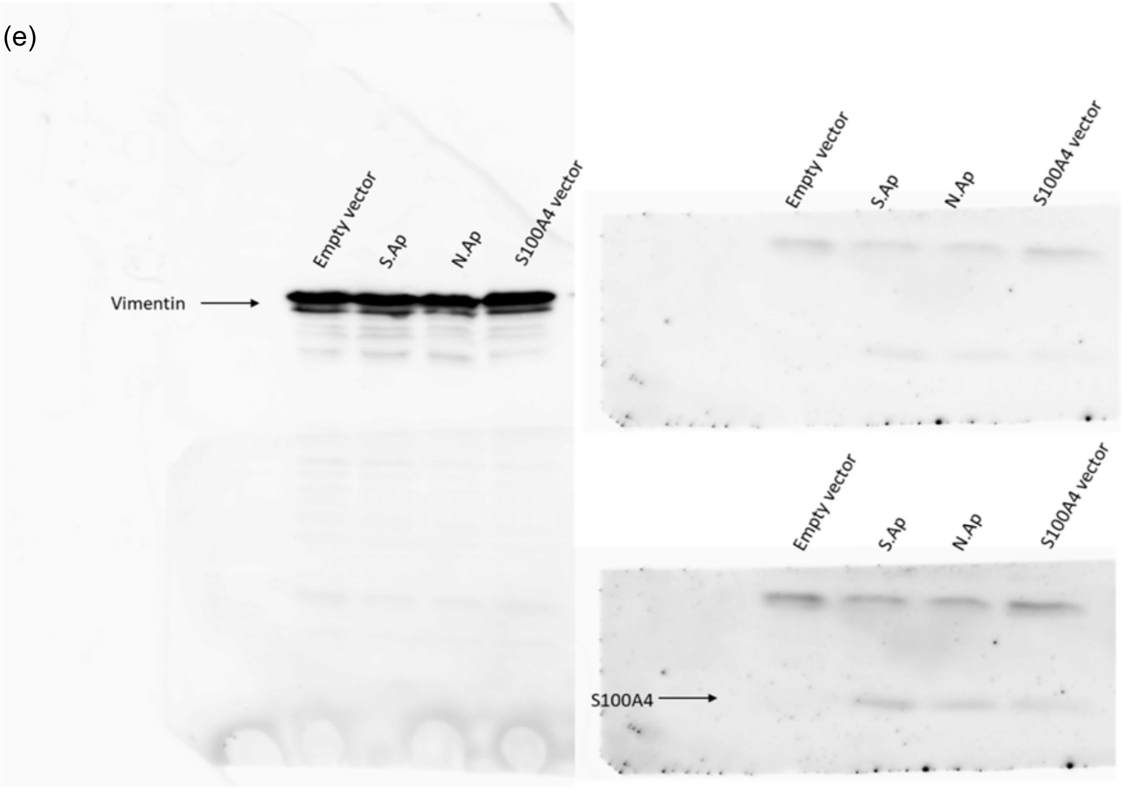


*Raw data of western blot showing α-SMA and S100A4 expression in transfected S-SMCs 96 hours after transfection with empty vector, S. Ap, and N. Ap. Positive control represents rhomboid smooth muscle cells. S100A4 vector represents SMCs transfected with an S100A4 expressing vector.*

*In some experiments, membranes were cut horizontally between 25kDa and 35kDa and incubated with anti-α-SMA or anti-vimentin (upper part of the membrane) or anti-S100A4 (lower part of the membrane) which explains the lack of full-length blots. All corresponding samples (α-SMA, vimentin or S100A4) derive from the same experiment and were processed in parallel.*
*(a,b,c) Membranes were incubated with the homemade mouse monoclonal IgM antibody recognizing S100A4. (d,e) Membranes were incubated with the mouse monoclonal IgG1 anti-S100A4 antibody AMAB90596, Sigma-Aldrich. In (e), the right panels show two different exposition times.*

**Figure 3C**

BrdU Staining:

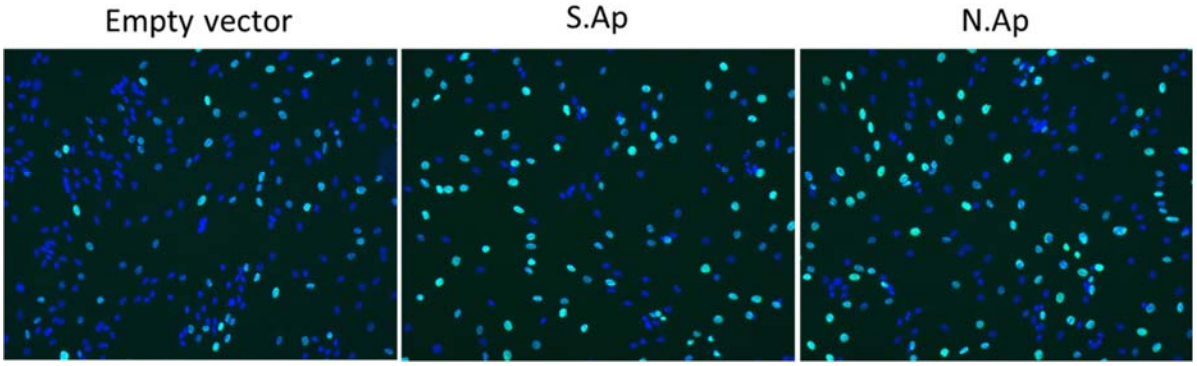


BrdU positive cell percentage quantification:

| | E vector | S. Ap | N. Ap |
|---|---|---|---|
| Exp 1 | 32.25 | 53.35 | 48.5 |
| Exp 2 | 31.2 | 54.54 | 58.62 |
| Exp 3 | 39 | 58.26 | 52.53 |

*Immunofluorescence staining showing BrdU-positive cells in transfected S-SMCs 96 hours after transfection with empty vector, S. Ap, and N. Ap and quantification of BrdU-positive cells percentage from 3 independent experiments.*

## Figure 3D

### S100A4 ELISA:

*Table representing experimental Optical density values and concentration of extracellular S100A4 detected by competitive ELISA assay in S-SMC supernatants after transfection with empty vector, S. Ap, and N. Ap from 6 independent experiments.*

| n=6 | OD1 | OD2 | OD3 | OD4 | OD5 | OD6 | OD Mean | Conc [nM] | Conc [pM] | Nb cells | concen/1000 cell | Normalized data |
|---|---|---|---|---|---|---|---|---|---|---|---|---|
| Empty vector | 0.131 | 0.126 | 0.137 | | | | 0.131 | 0.632 | 632 | 990000 | 0.638383838 | 1 |
| S.Ap | 0.102 | 0.079 | 0.137 | | | | 0.106 | 1.191 | 1191 | 990000 | 1.203030303 | 1.884493671 |
| N.Ap | 0.1 | 0.097 | 0.127 | | | | 0.108 | 1.133 | 1133 | 1026000 | 1.104288499 | 1.72981901 |
| | | | | | | | | | | | | |
| Empty vector | 0.311 | 0.326 | 0.317 | | | | 0.318 | 0.427 | 427.415 | 1038000 | 0.411767757 | 1 |
| S.Ap | 0.267 | 0.271 | 0.26 | | | | 0.266 | 0.819 | 818.731 | 840000 | 0.974679468 | 2.367061167 |
| N.Ap | 0.227 | 0.261 | 0.276 | | | | 0.255 | 0.943 | 943.335 | 852000 | 1.107201232 | 2.688897353 |
| | | | | | | | | | | | | |
| Empty vector | 0.279 | 0.276 | 0.261 | | | | 0.272 | 0.760 | 759.572 | 990000 | 0.767244569 | 1 |
| S.Ap | 0.214 | 0.233 | 0.238 | | | | 0.228 | 1.311 | 1311.057 | 906000 | 1.447082277 | 1.886076924 |
| N.Ap | 0.222 | 0.219 | 0.297 | | | | 0.221 | 1.446 | 1445.926 | 1026000 | 1.409284675 | 1.83681284 |
| | | | | | | | | | | | | |
| Empty vector | 0.289 | 0.278 | 0.294 | 0.286 | 0.268 | 0.295 | 0.285 | 0.235 | 234.570 | 990000 | 0.237 | 1 |
| S.Ap | 0.251 | 0.249 | 0.251 | 0.257 | 0.253 | 0.233 | 0.249 | 0.427 | 427.415 | 906000 | 0.471 | 1.987341772 |
| N.Ap | 0.238 | 0.256 | 0.268 | 0.246 | 0.247 | 0.25 | 0.251 | 0.415 | 414.553 | 1026000 | 0.404 | 1.70464135 |
| | | | | | | | | | | | | |
| Empty vector | 0.157 | 0.241 | 0.182 | | | | 0.170 | 0.209 | 209 | 855000 | 0.245 | 1 |
| S.Ap | 0.162 | 0.14 | 0.154 | | | | 0.152 | 0.328 | 328 | 848300 | 0.386 | 1.578673711 |
| N.Ap | 0.139 | 0.127 | 0.176 | | | | 0.147 | 0.369 | 369 | 902000 | 0.410 | 1.673409258 |
| | | | | | | | | | | | | |
| Empty vector | 0.152 | 0.186 | 0.167 | | | | 0.168 | 0.216 | 216 | 883000 | 0.244 | 1 |
| S.Ap | 0.15 | 0.134 | 0.122 | | | | 0.135 | 0.503 | 503 | 967500 | 0.519 | 2.127108662 |
| N.Ap | 0.122 | 0.147 | 0.136 | | | | 0.135 | 0.507 | 507 | 1020400 | 0.497 | 2.034145988 |

**Figure 4A**

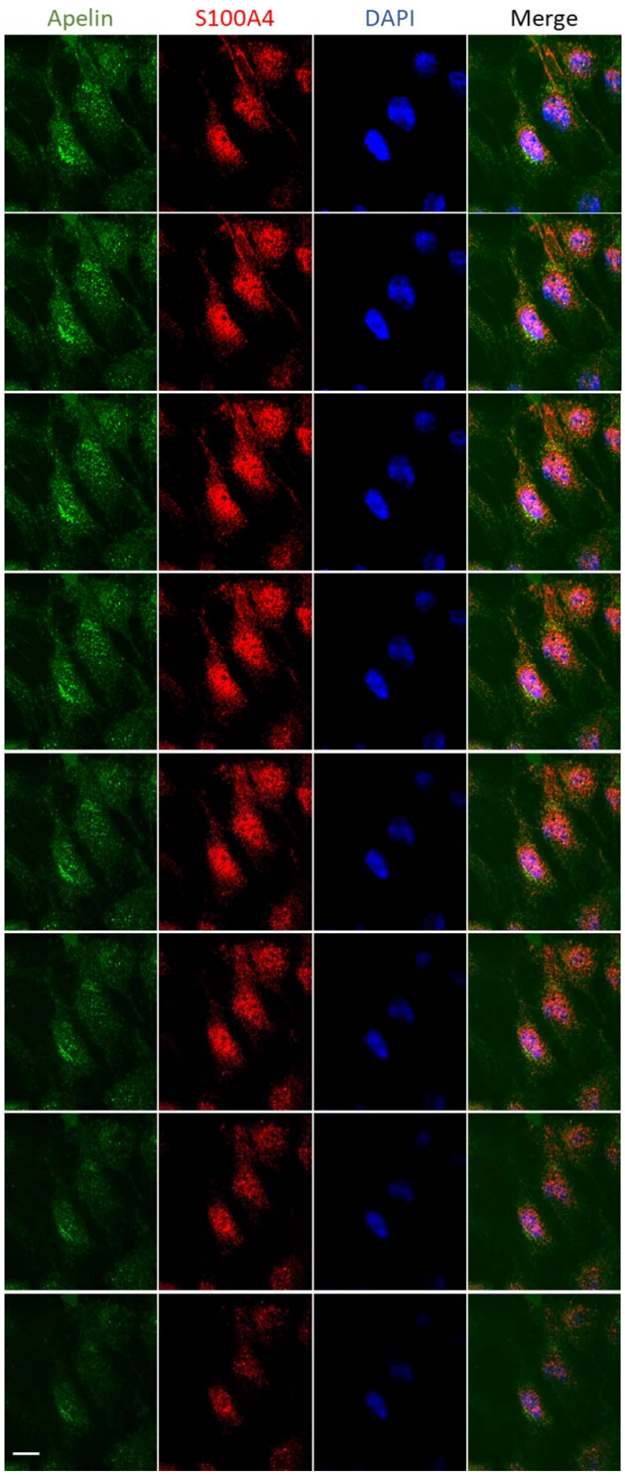


*Z-stack of confocal imaging of PDGF-BB stimulated SMCs showing apelin and S100A4 nuclear localization.*

## Figure 4B and C

| | | PDGF-BB treated SMCs | | | | | Control SMCs | | | |
|---|---|---|---|---|---|---|---|---|---|---|
| | Cell number | **Nuc Apelin** | **Nuc S100A4** | **Cyto Apelin** | **Cyto S100A4** | Cell number | **NucApelin** | **Nuc S100A4** | **Cyto Apelin** | **Cyto S100A4** |
| Values of CCD Unit/5sec | 1 | 474 | 120 | 264 | 127 | 1 | 172 | 121 | 188 | 117 |
| | 2 | 262 | 130 | 285 | 128 | 2 | 140 | 113 | 168 | 101 |
| | 3 | 263 | 163 | 296 | 128 | 3 | 204 | 138 | 189 | 123 |
| | 4 | 244 | 114 | 270 | 129 | 4 | 183 | 113 | 216 | 122 |
| | 5 | 243 | 196 | 231 | 123 | 5 | 170 | 101 | 207 | 111 |
| | 6 | 194 | 128 | 185 | 125 | 6 | 138 | 97 | 185 | 109 |
| | 7 | 215 | 161 | 222 | 123 | 7 | 143 | 109 | 179 | 107 |
| | 8 | 150 | 101 | 195 | 105 | 8 | 152 | 127 | 143 | 121 |
| | 9 | 162 | 103 | 140 | 100 | 9 | 111 | 112 | 116 | 103 |
| | 10 | 303 | 174 | 339 | 130 | 10 | 137 | 110 | 163 | 118 |
| | 11 | 272 | 127 | 312 | 137 | 11 | 119 | 104 | 180 | 165 |
| | 12 | 324 | 194 | 313 | 127 | 12 | 136 | 108 | 135 | 120 |
| | 13 | 312 | 259 | 265 | 332 | 13 | 188 | 100 | 194 | 99 |
| | 14 | 419 | 148 | 241 | 110 | 14 | 216 | 103 | 254 | 110 |
| | 15 | 245 | 154 | 215 | 134 | 15 | 181 | 105 | 222 | 112 |
| | 16 | 170 | 134 | 177 | 122 | 16 | 211 | 113 | 251 | 117 |
| | 17 | 172 | 110 | 181 | 104 | 17 | 166 | 106 | 152 | 100 |
| | 18 | 151 | 199 | 150 | 135 | 18 | 189 | 129 | 230 | 138 |
| | 19 | 200 | 223 | 204 | 141 | 19 | 156 | 134 | 224 | 150 |
| | 20 | 207 | 246 | 209 | 136 | 20 | 182 | 119 | 222 | 123 |
| | 21 | 106 | 107 | 107 | 107 | 21 | 311 | 176 | 361 | 198 |
| | 22 | 161 | 144 | 169 | 124 | 22 | 270 | 179 | 362 | 164 |
| | 23 | 130 | 125 | 129 | 120 | 23 | 274 | 149 | 307 | 145 |
| | 24 | 116 | 116 | 122 | 107 | 24 | 352 | 158 | 292 | 127 |
| | 25 | 103 | 100 | 122 | 105 | 25 | 686 | 161 | 419 | 156 |
| | 26 | 358 | 501 | 307 | 435 | 26 | 533 | 147 | 475 | 147 |
| | 27 | 323 | 108 | 337 | 164 | 27 | 332 | 143 | 501 | 146 |
| | 28 | 458 | 262 | 402 | 167 | 28 | 149 | 128 | 286 | 139 |
| | 29 | 442 | 205 | 320 | 162 | 29 | 253 | 126 | 241 | 122 |
| | 30 | 208 | 165 | 253 | 128 | 30 | 216 | 104 | 191 | 110 |
| | 31 | 381 | 315 | 353 | 193 | 31 | 256 | 137 | 398 | 185 |
| | 32 | 278 | 329 | 358 | 159 | 32 | 242 | 184 | 270 | 167 |
| | 33 | 374 | 318 | 356 | 174 | 33 | 221 | 115 | 137 | 119 |
| | 34 | 396 | 284 | 367 | 184 | | | | | |
| | 35 | 309 | 320 | 358 | 182 | | | | | |
| | 36 | 229 | 197 | 294 | 146 | | | | | |
| | 37 | 266 | 169 | 299 | 153 | | | | | |
| | 38 | 573 | 187 | 428 | 184 | | | | | |
| | 39 | 553 | 178 | 397 | 196 | | | | | |
| | 40 | 229 | 178 | 287 | 153 | | | | | |

*Table representing CCD unit values of Nuclear apelin, nuclear S100A4, cytoplasmic apelin, cytoplasmic S100A4 for each analysed cell treated with PDGF-BB vs Control.*

# Figure 4D and E

Proximity Ligation Assay:

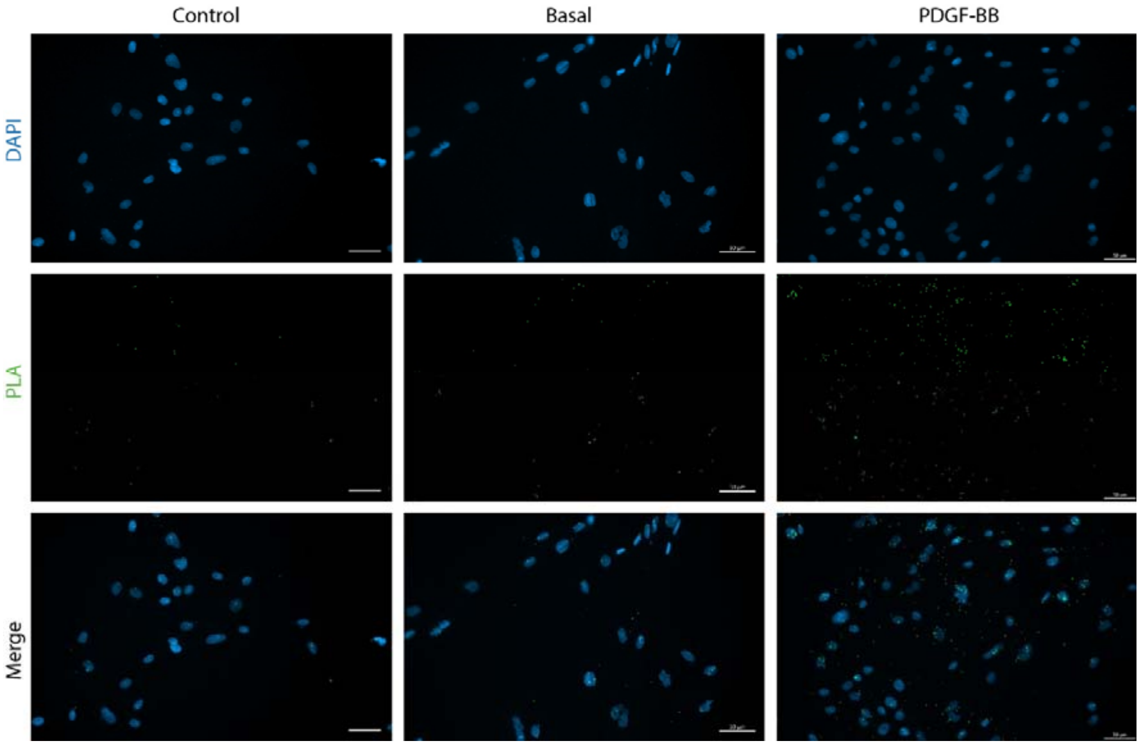


| n=4 | Number of Cells | Dots | Dots/cell |
|---|---|---|---|
| Ctrl | 262 | 182 | 0.6946565 |
| Basal | 275 | 480 | 1.7454545 |
| PDGF-BB | 108 | 518 | 4.7962963 |
| | | | |
| Ctrl | 192 | 96 | 0.5 |
| Basal | 304 | 586 | 1.9276316 |
| PDGF-BB | 52 | 227 | 4.3653846 |
| | | | |
| Ctrl | 257 | 95 | 0.3696498 |
| Basal | 61 | 151 | 2.4754098 |
| PDGF-BB | 79 | 297 | 3.7594937 |
| | | | |
| Ctrl | 108 | 73 | 0.6759259 |
| Basal | 36 | 55 | 1.5277778 |
| PDGF-BB | 164 | 796 | 4.8536585 |

*Proximity ligation assay showing the interaction between apelin and S100A4 upon PDGF-BB stimulation. Representative field image with quantification of PLA dots/cell from 4 independent biological replicates*

**Figure 5C**

Green Fluorescent Protein (GFP) Western Blots:

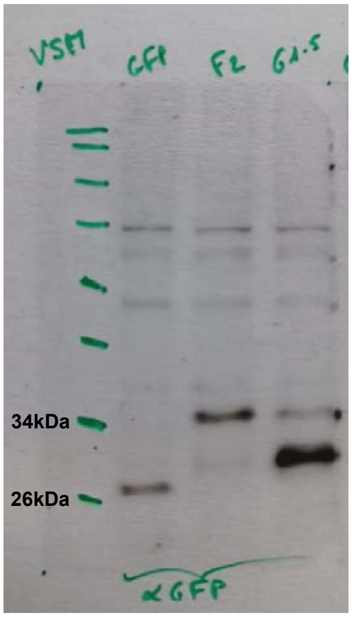


*Raw data of western blot showing GFP expression in transfected S-SMCs 24 hours after transfection with empty GFP vector (pEGFP-N1, GFP), S. Ap (F2), and N. Ap (G1.5).*
